# Laser‐ und energiebasierte Systeme zur Behandlung der Rosazea – ein systematischer Review mit Netzwerk‐Metaanalyse

**DOI:** 10.1111/ddg.15961_g

**Published:** 2026-01-14

**Authors:** Lynhda Nguyen, Christina Sorbe, Nikolaus Seeber, Stefan W. Schneider, Katharina Herberger

**Affiliations:** ^1^ Abteilung für Laser und Ästhetik Klinik und Poliklinik für Dermatologie and Venerologie Universitätsklinikum Hamburg‐Eppendorf, Hamburg; ^2^ nstitut für Versorgungsforschung in der Dermatologie und bei Pflegeberufen Universitätsklinikum Hamburg‐Eppendorf, Hamburg; ^3^ Hautarztpraxis Dres. Peter/Seeber/Altheide, Hamburg; ^4^ Klinik und Poliklinik für Dermatologie and Venerologie Universitätsklinikum Hamburg‐Eppendorf, Hamburg

**Keywords:** IPL, Laser, Netzwerk‐Metaanalyse, nichtionisierende Strahlquellen, Rosazea, Energy‐based devices, IPL, laser, network meta‐analysis, rosacea

## Abstract

**Hintergrund und Zielsetzung:**

Laser und energiebasierte Geräte (energy‐based devices, EBDs) werden häufig zur Behandlung vaskulärer Symptome eingesetzt, jedoch fehlen umfassende Vergleichsdaten. Ziel dieser Studie war es, Wirksamkeit und Sicherheit verschiedener laser‐ und energiebasierter Systeme bei Rosazea in einem systematischen Review mit Netzwerk‐Metaanalyse (NMA) zu vergleichen.

**Methoden:**

Randomisierte kontrollierte Studien (RCTs) wurden aus MEDLINE, CENTRAL, Web of Science und Registern laufender Studien identifiziert. Primäre Endpunkte waren Patientenzufriedenheit, Verbesserung von Erythem und Teleangiektasien sowie langfristige unerwünschte Ereignisse (UE). Zusätzlich wurden das Verzerrungsrisiko (Risk of Bias, RoB) und ein möglicher Publikationsbias analysiert.

**Ergebnisse:**

25 RCTs wurden in die qualitative Analyse einbezogen; ein Teil floss in die NMA ein. Meist lag ein unklares bis hohes Bias‐Risiko vor. Die Radiofrequenz‐Microneedling‐Therapie war der gepulsten Farbstofflasertherapie (FSL) in Bezug auf die Patientenzufriedenheit (MD –1,32; 95%‐KI –1,89 bis –0,76) und das Erythem (MD –1,44; 95%‐KI –1,96 bis –0,91) überlegen. Für die Behandlung von Teleangiektasien erwies sich die Kombination aus Oxymetazolin und FSL als vorteilhaft (MD –0,58; 95%‐KI –1,03 bis –0,14). Nebenwirkungs‐ und Abbruchraten waren zwischen den verschiedenen Therapien vergleichbar.

**Schlussfolgerungen:**

Diese erste NMA zu Lasern und EBDs bei Rosazea zeigt die Notwendigkeit robuster Studien, insbesondere zu Langzeit‐ und Kombinationstherapien.

## EINLEITUNG

Die Rosazea ist eine chronisch‐entzündliche Hauterkrankung, die vor allem die zentralen Gesichtspartien betrifft. Die berichtete Prävalenz variiert zwischen den Studien und reicht von unter 1% bis zu 22%.^1^ Die Erkrankung weist verschiedene Phänotypen auf, von einem transienten und persistierenden Erythem über Teleangiektasien und entzündliche Läsionen bis hin zu phymatösen Veränderungen.^2^ Zusätzlich können Patienten Beschwerden wie Juckreiz oder Brennen verspüren. Das auffällige Erscheinungsbild der Rosazea kann zudem das Selbstwertgefühl beeinträchtigen und sich negativ auf die Lebensqualität der Betroffenen auswirken.^3^

Zur Behandlung der Rosazea stehen verschiedene Optionen zur Verfügung, darunter topische und systemische Medikamente, Laser‐ und energiebasierte Geräte (energy‐based devices, EBDs) sowie Kombinationstherapien, insbesondere bei fortgeschrittenen Verläufen.^4^ In den letzten Jahren haben sich Laser‐ und energiebasierte Therapien als bewährte Methoden zur Behandlung der Rosazea etabliert.^4^ Diese Verfahren beruhen auf dem Prinzip der selektiven Photothermolyse, bei dem bestimmte Wellenlängen ausschließlich von den entsprechenden Zielchromophoren absorbiert werden.^5^ Bei der Rosazea dienen Hämoglobin und Oxyhämoglobin als Zielchromophore: Hämoglobin weist Absorptionsspitzen bei 432 nm und 556 nm auf, während Oxyhämoglobin Absorptionsspitzen bei 414 nm, 542 nm und 576 nm hat.^6^

Unter den Lasern und EBDs gelten gepulste Farbstofflaser (FSL) derzeit als Methode der Wahl zur Behandlung der Rosazea. Angesichts der Vielzahl verfügbarer Systeme zur Therapie gefäßbasierter Erkrankungen ist ein umfassender Vergleich erforderlich. Konventionelle Metaanalysen stoßen jedoch an ihre Grenzen, wenn es darum geht, mehrere Behandlungen gleichzeitig zu bewerten. Eine Netzwerk‐Metaanalyse (NMA), die sowohl direkte als auch indirekte Vergleiche ermöglicht, bietet hierfür eine Lösung.

Nach unserem Kenntnisstand gibt es bislang keine Studien, die eine vergleichende Netzwerkanalyse energie‐ und laserbasierter Therapien zur Behandlung der Rosazea erstellt haben. Ziel dieser Studie ist es daher, die Wirksamkeit, Sicherheit und Verträglichkeit aller verfügbaren Laser‐ und energiebasierten Behandlungen für die Rosazea systematisch zu evaluieren.

## MATERIAL UND METHODIK

### Protokoll und Registrierung

Eine systematische Übersichtsarbeit und Netzwerk‐Metaanalyse (NMA) randomisierter kontrollierter Studien (RCTs) zu laser‐ und energiebasierten Behandlungen der Rosazea wurde gemäß den erweiterten PRISMA‐Richtlinien (Preferred Reporting Items for Systematic Reviews and Meta‐Analyses) durchgeführt.^7^, ^8^ Gemäß dem PICO‐Schema bestand die *Population* aus Rosazea‐Patienten. *Intervention* und *Vergleichsintervention* waren Laser‐ und energiebasierte Behandlungen. Die *Endpunkte* umfassten die Patientenzufriedenheit, vom Untersucher bewertete Reduktion von Erythem und Teleangiektasien sowie kurz‐ und langfristige unerwünschte Ereignisse (UE). Die Studienauswahl, Datenextraktion und Bewertung des Verzerrungsrisikos (RoB) wurden unabhängig voneinander durch zwei Autoren (L.N., K.H.) durchgeführt. Bei Unstimmigkeiten wurde ein dritter Gutachter (S.W.S.) hinzugezogen. Das Studienprotokoll wurde vorab bei PROSPERO registriert (CRD42023485884).

### Suchstrategie

Wir durchsuchten bis zum 23. November 2023 die folgenden Datenbanken: MEDLINE (Ovid), Cochrane Central Register of Controlled Trials (CENTRAL) und Web of Science. Zusätzlich wurden die Studienregister WHO Trials Registry und Clinicaltrials.gov bis zum 25. Dezember 2023 durchsucht. Es wurden keine Sprachbeschränkungen bei der Suche angewendet. Die detaillierte Suchstrategie ist im Online‐Supplement S1 beschrieben. Darüber hinaus wurde bei allen eingeschlossenen Studien eine Vorwärts‐ (Zitationen) und Rückwärtssuche (Referenzen) mithilfe von Google Scholar und Web of Science durchgeführt. Alle identifizierten Studien wurden anhand von Titel und Abstract gescreent.

### Einschlusskriterien

Randomisierte kontrollierte Studien, die jegliche Formen von laser‐ oder energiebasierten Interventionen zur Behandlung von Rosazea untersuchten, wurden eingeschlossen. Studien, die sich auf Teleangiektasien im Gesicht konzentrierten, wurden ebenfalls eingeschlossen. Studien, die sich auf Rhinophym, okuläre Rosazea oder Morbus Morbihan bezogen, wurden ausgeschlossen, da es sich hierbei um Subtypen der Rosazea handelt.^9^ Studien, die lediglich eine einzelne Intervention untersuchten, wurden als Ein‐Gruppen‐Studien gewertet und daher nicht in die quantitative Analyse aufgenommen.

### Datenextraktion

Folgende Daten wurden extrahiert: Name des Erstautors, Veröffentlichungsjahr, Anzahl der Teilnehmenden, Geschlecht und Alter der Teilnehmenden, Fitzpatrick‐Hauttypen, Rosazea‐Phänotypen, Interventionen, Endpunkte, kurz‐ und langfristige UEs sowie Studienabbrüche aufgrund von UE.

### Primäre und sekundäre Endpunkte

Die primären Endpunkte umfassten die Patientenzufriedenheit, Verbesserungen von Erythem und Teleangiektasien sowie langfristige UEs wie postinflammatorische Hyperpigmentierung und Narbenbildung, die die Lebensqualität beeinträchtigen können. Zudem wurden Studienabbrüche aufgrund von UE berücksichtigt. Sekundäre Endpunkte waren kurzfristige UE wie Krustenbildung, Schwellungen und Erythem. Purpura wurde dabei nicht als Nebenwirkung gewertet, da sie den klinischen Zielpunkt mehrerer Lasertherapien darstellt.

### Bewertung des Verzerrungsrisikos

Das Verzerrungsrisiko (Risk of Bias; RoB) wurde mithilfe des Cochrane‐RoB‐Instruments für randomisierte Studien (RoB 2) bewertet.^10^ Dieses Instrument umfasst folgende Bereiche: *(1)* Generierung der Zufallssequenz, *(2)* Geheimhaltung der Zuteilung, *(3)* Verblindung von Teilnehmenden und Studienpersonal, *(4)* Verblindung der Ergebniserhebung, *(5)* unvollständige Ergebnisdaten, *(6)* selektive Berichterstattung sowie *(7)* sonstige Verzerrungen. Jeder Bereich konnte als „niedriges Risiko“, „hohes Risiko“ oder „unklares Risiko“ für eine Verzerrung eingestuft werden.^10^

### Publikationsbias

Ein möglicher Publikationsbias wurde anhand eines Funnel‐Plots, des Egger‐Tests sowie einer nichtparametrischen Trim‐and‐Fill‐Methode unter Verwendung des *metafor*‐Pakets in R untersucht.^11^, ^12^

### Statistische Analyse

Die Datenanalyse wurde mithilfe von Review Manager 5 (Cochrane Collaboration) durchgeführt. Eine Metaanalyse bewertete die Wirksamkeit der Läsionsreduktion im Vergleich zum 595 nm FSL unter Verwendung von Mittelwertdifferenzen (MD) mit 95%‐Konfidenzintervallen (KI) und Random‐Effects‐Modellen. Medianwerte und Interquartilsabstände, wie sie häufig in den Studien berichtet wurden, wurden mithilfe der Box‐Cox‐Methode in arithmetische Mittelwerte und Standardabweichungen umgerechnet.^13^, ^14^ Statistische Berechnungen erfolgten in R (Version 4.3.1) unter Verwendung des *netmeta*‐Pakets (Version 2.8.2) sowie des *estmeansd*‐Pakets (Version 1.0.0).^13^, ^14^, ^15^, ^16^ Ein additives NMA‐Modell wurde verwendet, um Behandlungseffekte zu bewerten und Inkonsistenzen (I^2^) in der NMA zu ermitteln.^17^ p‐Werte < 0,05 wurden als statistisch signifikant gewertet.

## ERGEBNISSE

### Suchergebnisse

**ABBILDUNG 1:**
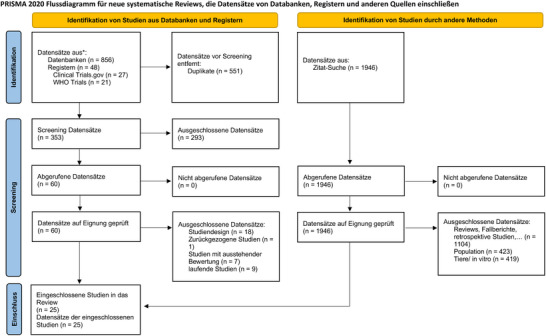
Flussdiagramm der Studie.

Die initiale systematische Literaturrecherche ergab 856 Publikationen und 48 Studienregistrierungen. Durch eine Vorwärts‐ und Rückwärtssuche wurden zusätzlich 1946 potenzielle Studien identifiziert. Nach Entfernung von Duplikaten und dem Screening der potenziellen Einträge anhand von Titel und Abstract verblieben 60 Artikel zur weiteren Bewertung. Nach weiterer Prüfung wurden 25 Studien in die qualitative Analyse eingeschlossen.^18^, ^19^, ^20^, ^21^, ^22^, ^23^, ^24^, ^25^, ^26^, ^27^, ^28^, ^29^, ^30^, ^31^, ^32^, ^33^, ^34^, ^35^, ^36^, ^37^, ^38^, ^39^, ^40^, ^41^, ^42^ 16 davon wurden in die quantitative Analyse aufgenommen (Abbildung 1).^18^, ^19^, ^20^, ^22^, ^24^, ^25^, ^26^, ^28^, ^31^, ^32^, ^33^, ^34^, ^35^, ^36^, ^38^, ^42^ Detaillierte Informationen zu den ein‐ und ausgeschlossenen Studien finden sich in den Online‐Supplementen S2 und S3.

### Studiencharakteristika

Die 25 RCTs, die in die qualitative Analyse eingeschlossenen wurden, umfassten insgesamt 843 Patienten mit einem Durchschnittsalter von 46,9 Jahren (18–85 Jahre). Für 48 Teilnehmende fehlten Angaben zum Geschlecht.^39^, ^42^ Unter den berichteten demografischen Daten waren 66,3% weiblich. In 14 Publikationen wurde der Fitzpatrick‐Hauttyp nicht angegeben.^24^, ^25^, ^27^, ^31^, ^35^, ^36^, ^38^, ^39^, ^40^ Bei den übrigen Studien verteilten sich die Hauttypen wie folgt: 8,3% Hauttyp I, 36,6% Hauttyp II, 32,2% Hauttyp III, 20% Hauttyp IV und 2,9% Hauttyp V. Patient:innen mit Hauttyp VI wurden in keiner Studie eingeschlossen. Von den eingeschlossenen Studien befassten sich 76% (insgesamt 690 Patienten) mit einem persistierendenb Erythem.^18^, ^19^, ^20^, ^21^, ^24^, ^25^, ^26^, ^27^, ^28^, ^29^, ^30^, ^31^, ^32^, ^33^, ^34^, ^37^, ^38^, ^40^, ^41^. In 88% der Studien (insgesamt 784 Patienten) wurden Teleangiektasien behandelt.^18^, ^19^, ^20^, ^21^, ^22^, ^23^, ^25^, ^26^, ^27^, ^28^, ^29^, ^30^, ^31^, ^32^, ^33^, ^34^, ^35^, ^36^, ^38^, ^39^, ^40^, ^41^, ^42^ Ausführliche Informationen finden sich im Online‐Supplement S2.

### Qualitätsbewertung

**ABBILDUNG 2:**
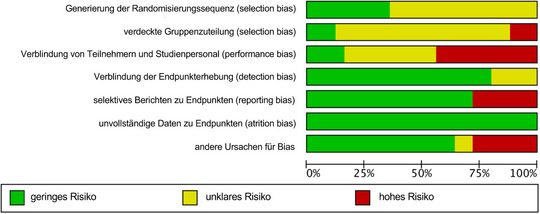
Grafik zum Verzerrungsrisiko: Übersicht der Einschätzungen der Autoren zu jedem Bereich des Cochrane‐Verzerrungsrisiko‐Instruments, dargestellt als prozentuale Verteilung aller eingeschlossenen Studien. Grün (+): geringes Verzerrungsrisiko; Gelb (±): unklar; Rot (–): hohes Verzerrungsrisiko.

**ABBILDUNG 3:**
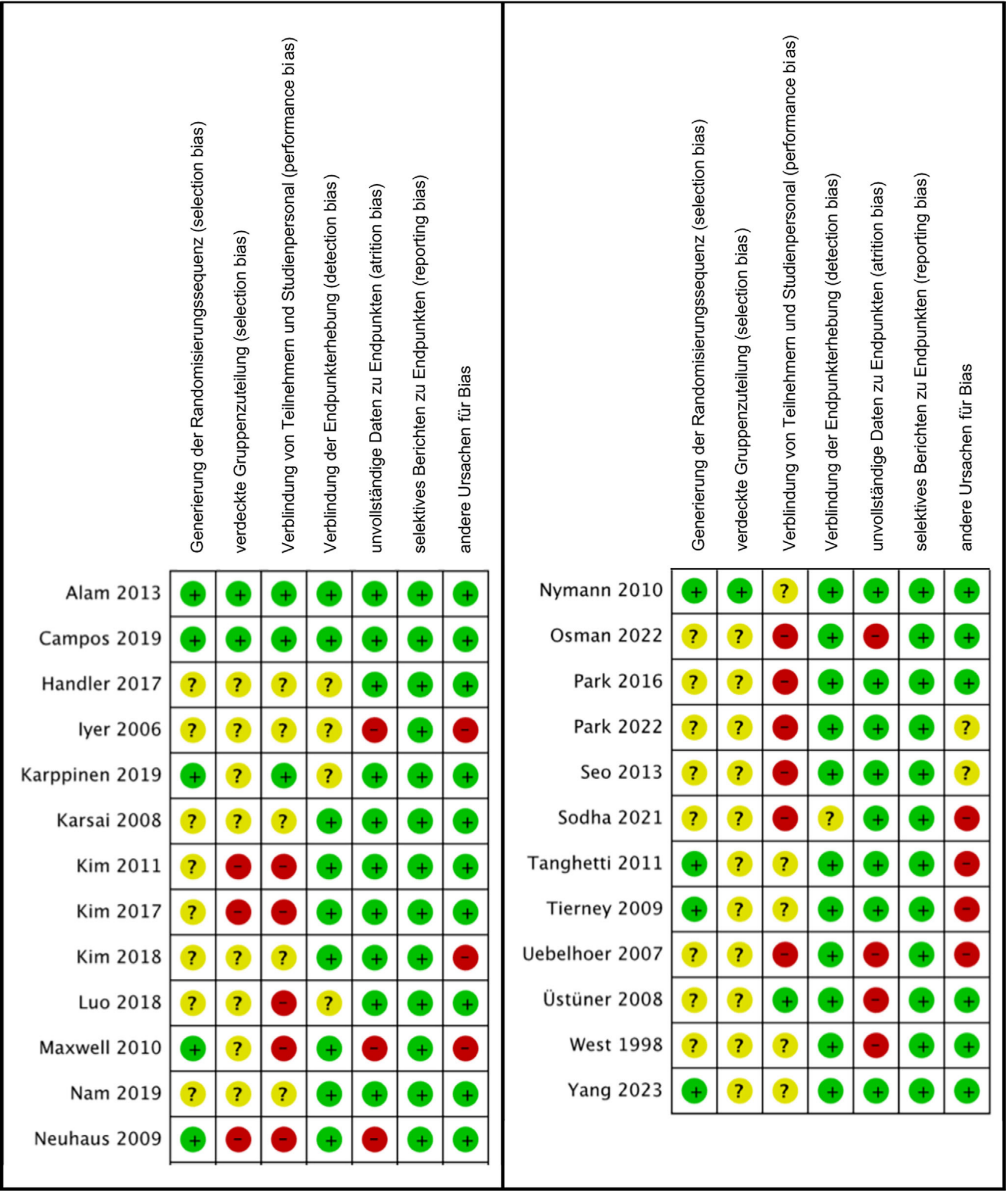
Zusammenfassung der Bewertungen der Autoren zu jedem Bereich des Cochrane‐Verzerrungsrisiko‐Instruments für jede der eingeschlossenen Studien.

Die meisten Studien zeigten in mindestens einem Bereich ein unklares RoB, hauptsächlich aufgrund unzureichender Berichterstattung (Abbildungen 2, 3). Das Risiko im Zusammenhang mit der Verblindung der Durchführung sowie mit dem Selektionsbias wurde häufig als unklar oder hoch eingestuft, was vor allem auf die Natur gerätebasierter Behandlungen zurückzuführen ist. Detaillierte Analysen zum RoB der eingeschlossenen Studien finden sich im Online‐Supplement S2.

Bei der Auswertung zur Patientenzufriedenheit zeigte die Funnel‐Plot‐Analyse eine gleichmäßige Verteilung der Ergebnisse mit einem Ausreißer (RFMN). Der Egger‐Test (p < 0,1925) wies nicht auf einen Publikationsbias hin. Die Trim‐and‐Fill‐Methode ergänzte keine weiteren Studien. Für das Erythem zeigte der Funnel‐Plot eine ebenfalls gleichmäßige Verteilung mit einem Ausreißer (RFMN). Der Egger‐Test (p < 0,150) deutete auf keinen signifikanten Bias hin, und die Trim‐and‐Fill‐Methode ergänzte eine Studie, was auf einen minimalen Publikationsbias hinweist.

Bezüglich der Teleangiektasien wies der Funnel‐Plot zwei Ausreißer (IPL; Niacin + FSL) auf. Ansonsten war die Verteilung symmetrisch. Der Egger‐Test (p < 0,149) sowie die Ergänzung von drei Studien durch die Trim‐and‐Fill‐Methode deuten auf einen moderaten Publikationsbias hin. Weitere Details finden sich im Online‐Supplement S4.

### Behandlungsergebnisse

**ABBILDUNG 4:**
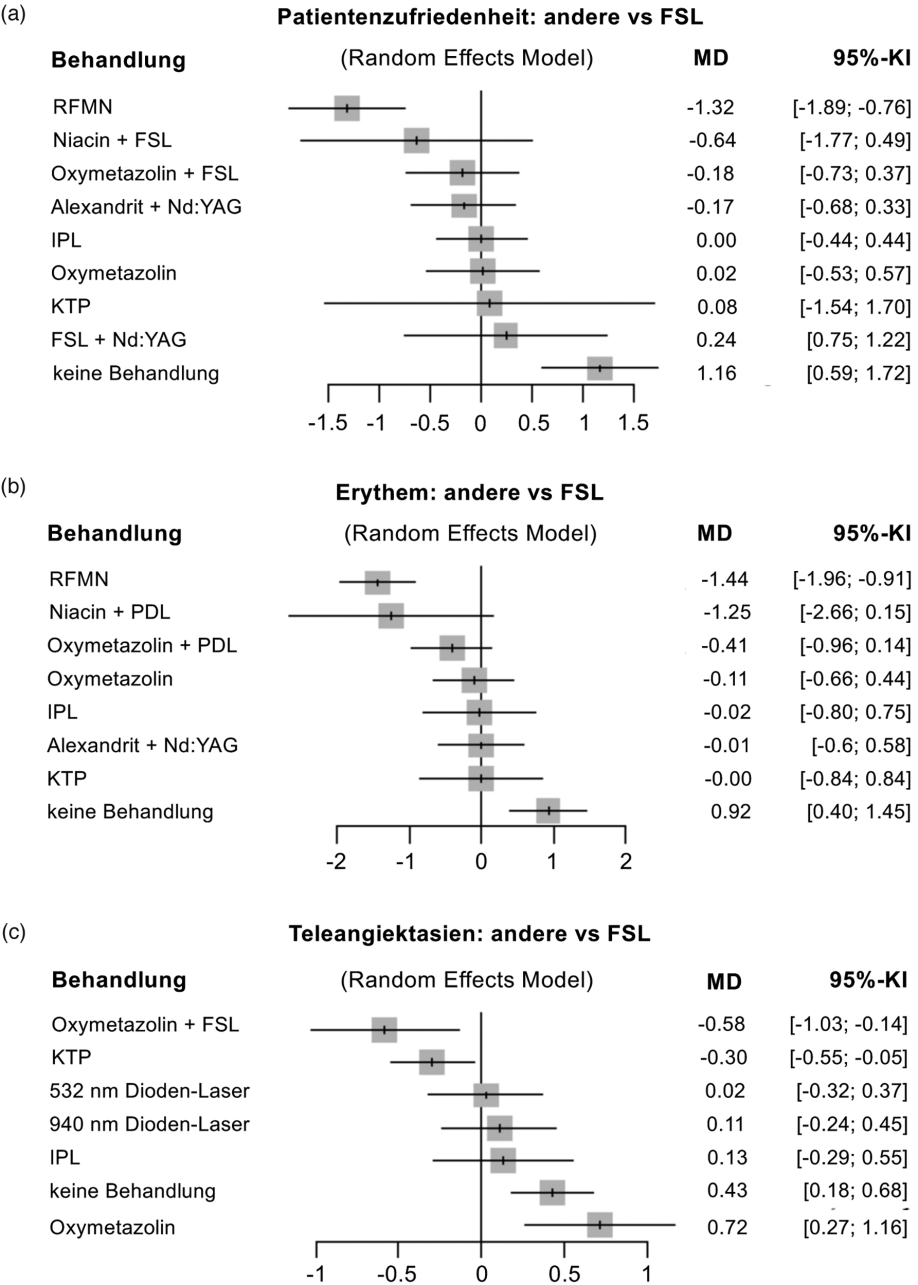
Netzwerk‐Metaanalyse zur (a) Patientenzufriedenheit, (b) Wirksamkeit in der Behandlung von Erythem sowie (c) Teleangiektasien bei Rosazea mit verschiedenen Laser‐ und energiebasierten Verfahren im Vergleich zum gepulsten Farbstofflaser (FSL). Ein mittlerer Unterschied (MD) von < 0 weist auf eine höhere Wahrscheinlichkeit für eine effektivere Behandlung der Vergleichsmethode hin. *Abk*.: MD, mittlerer Unterschied; KI, Konfidenzintervall; RFMN, Radiofrequenz‐Microneedling; IPL, intensives gepulstes Licht; Nd:YAG, Neodym‐dotierter Yttrium‐Aluminium‐Granat; KTP, Kaliumtitanylphosphat.

Zur besseren Vergleichbarkeit der unterschiedlichen Bewertungsmethoden in den eingeschlossenen Studien wurden alle Daten auf eine einheitliche visuelle Analogskala von 1 bis 5 (gering bis stark ausgeprägt) normiert, da diese Skala am häufigsten verwendet wurde. Die Patientenzufriedenheit sowie die Wirksamkeit von Lasern und EBDs in der Behandlung von Erythem und Teleangiektasien im Vergleich zur FSL‐Monotherapie sind in den Forest‐Plots dargestellt (Abbildung 4). Die Therapieansätze sind entsprechend ihrer Wirksamkeit, von der effektivsten bis zur am wenigsten effektiven Methode, geordnet.

#### Patientenzufriedenheit

Für die Analyse der Patientenzufriedenheit wurden sieben Studien in die quantitative Analyse einbezogen. Die NMA umfasste sienben paarweise Vergleiche mit zehn verschiedenen Behandlungsansätzen und beinhaltete 321 Beobachtungen sowie drei Subnetzwerke (Abbildung 4, Online‐Supplement S5). Das RFMN (MD –1,32; 95%‐KI –1,89 bis –0,76) erwies sich als signifikant wirksamer bei der Reduktion des Erythems bei Rosazea, gefolgt von Niacin + FSL (MD –0,64; 95%‐KI –1,77 bis 0,49) und Oxymetazolin + FSL (MD –0,18; 95%‐KI –0,73 bis 0,37). Die am wenigsten vielversprechende Strategie war keine Behandlung (MD 1,16; 95%‐KI 0,59 bis 1,72).

#### Erythem

Insgesamt wurden acht Studien in die quantitative Analyse zur Verbesserung des Erythems einbezogen. Die NMA umfasste acht paarweise Vergleiche mit neun unterschiedlichen Therapieansätzen, basierend auf 272 Beobachtungen und 3 Subnetzwerken (Abbildung 4, Online‐Supplement S5).

Das RFMN (MD –1,44; 95%‐KI –1,96 bis –0,91) zeigte eine deutliche Wirksamkeit bei der Behandlung des Erythems bei Rosazea. Im Gegensatz dazu stellte sich keine Behandlung als die am wenigsten wirksame Methode heraus (MD 0,92; 95%‐KI 0,40 bis 1,45).

#### Telangiektasien

In die NMA zur Behandlung von Teleangiektasien wurden fünf Studien in die quantitative Analyse einbezogen. Diese umfasste fünf paarweise Vergleiche über acht verschiedene Therapieansätze mit insgesamt 177 Beobachtungen und vier Subnetzwerken (Abbildung 4, Online‐Supplement S5). Oxymetazolin + FSL erwies sich als signifikant wirksamer in der Behandlung von Teleangiektasien bei Rosazea (MD –0,58; 95%‐KI –1,03 bis –0,14), gefolgt vom KTP‐Laser (MD –0,30; 95%‐KI –0,55 bis –0,05). Die am wenigsten wirksamen Behandlungen waren Oxymetazolin allein (MD 0,72; 95%‐KI 0,27 bis 1,16) sowie keine Behandlung (MD 0,43; 95%‐KI 0,18 bis 0,68).

#### Unerwünschte Ereignisse

Alle in die Analyse eingeschlossenen Studien berichteten über UE. Jedoch lieferten nur sechs Studien Angaben zur Häufigkeit kurzfristiger Nebenwirkungen, darunter posttherapeutisches Erythem, Schwellung, Krustenbildung und Blasenbildung, die sich jeweils innerhalb weniger Tage zurückbildeten.^19^, ^20^, ^24^, ^26^, ^32^, ^36^ Langfristige Nebenwirkungen wurden in 15 Studien dokumentiert.^20^, ^22^, ^24^, ^26^, ^27^, ^28^, ^29^, ^31^, ^32^, ^33^, ^34^, ^35^, ^36^, ^40^, ^41^ Weitere Einzelheiten finden sich im Online‐Supplement S2.

Aufgrund unvollständiger Daten konnte keine Metaanalyse zu UE durchgeführt werden. Karppinen et al. (2019) berichteten über oberflächliche atrophische Narben bei 11,1% der Patient:innen, die mit dem 585 nm Gelblichtlaser behandelt wurden.^22^ Luo et al. (2020) beobachteten bei 10,8% der IPL‐behandelten Fälle langfristige Nebenwirkungen, darunter Gesichtsbrennen, Hyperpigmentierung und Blasenbildung.^27^ Darüber hinaus dokumentierten Seo et al. (2013) post‐therapeutische Hyperpigmentierung bei je einem Patienten unter IPL‐ (1/18) und FSL‐Behandlung (1/19).^33^

### Therapieabbrüche aufgrund von unerwünschten Ereignissen

Aufgrund fehlender signifikanter Unterschiede zwischen den Behandlungen wurde keine Metaanalyse durchgeführt. Alam et al. (2013) berichteten, dass zwei von 16 Patienten die Studie wegen posttherapeutischer Schwellung abbrachen, wobei unklar blieb, ob die Reaktion nach einer FSL‐ oder Nd:YAG‐Behandlung auftrat.^18^ Ebenso vermerkten Campos et al. (2019) zwei Abbrüche unter 29 Patienten – einer aufgrund inakzeptabler Purpura, ein weiterer wegen starker Schmerzen, ohne Angabe der erfolgten Laserbehandlung.^19^ Seo et al. (2016) beobachteten einen Studienabbruch unter 25 mit FSL behandelten Patienten wegen Verschlechterung der Symptome.^33^ Üstüner et al. (2018) berichteten über zwei Abbrüche unter 30 Patienten, die mit KTP‐ oder Nd:YAG‐Lasern behandelt wurden.^41^

In der Studie von Luo et al. (2020) zeigten vier von 130 unbehandelten Kontrollpatienten, die keine Behandlung erhielten, eine Verschlechterung der Rosazea, während sechs von 130 IPL‐behandelten Patienten die Studie aufgrund von Blasen, Brennen, Rötung oder Ödem abbrachen.^27^ Auch Neuhaus et al. berichteten, dass ein IPL‐behandelter Patient (1/30) wegen übermäßiger Schwellung abbrach, wobei eine weitere Auswertung der UE nicht möglich war, da der Nachsorgetermin nicht stattfand.^29^

Darüber hinaus brach ein Patient (1/18), der mit einer Kombination aus FSL und Oxymetazolin behandelt wurde, die Studie aus nicht näher genannten Gründen ab, ebenso ein weiterer (1/16), der nur topisches Oxymetazolin erhielt.^34^ Schließlich berichteten Yang et al. (2023) über einen Studienabbruch unter 22 mit Minocyclin (100 mg) behandelten Patienten, infolge von Nebenwirkungen.^40^

## DISKUSSION

Diese systematische Übersichtsarbeit mit NMA bietet eine vergleichende Analyse der Wirksamkeit, Sicherheit und Verträglichkeit verschiedener laser‐ und energiebasierter Behandlungen bei Rosazea auf Basis der bislang veröffentlichten RCTs.

Im Gegensatz zu systemischen und topischen medikamentösen Therapien zeigt unsere Analyse, dass die Evidenzlage zur Anwendung von Lasern und EBDs bei Rosazea bislang relativ unklar ist. Übereinstimmend weisen aktuelle Übersichtsarbeiten auf eine Evidenz mit niedriger bis mittlerer Sicherheit hin, dass FSL, Nd:YAG‐Laser und IPL eine Reduktion des Hintergrunderythems und der Teleangiektasien bei Rosazea‐Patienten bewirken können.^4^, ^43^ Diese NMA berücksichtigte auch unkonventionelle Therapieansätze wie die Kombination von Oxymetazolin und FSL sowie die RFMN‐Therapie. Beide Methoden zeigten vielversprechende Ergebnisse in Bezug auf die Patientenzufriedenheit und die Linderung der Rosazeasymptome. Die Funnel‐Plot‐Analyse identifizierte RFMN jedoch als Ausreißer in diesen Kategorien, was auf ein mögliches Publikationsbias hindeutet. Insbesondere die Kombination aus FSL und topischem Oxymetazolin wies in unserer Analyse die höchste relative Wirksamkeit bei der Behandlung Rosazea‐assoziierter Teleangiektasien auf. Diese Bewertung basiert jedoch ausschließlich auf den verfügbaren RCT‐Daten und spiegelt möglicherweise nicht die breitere klinische Erfahrung und Langzeitergebnisse wider. KTP‐Laser sind hingegen seit Langem in der Praxis als wirksame und gut verträgliche Therapie etabliert mit hohen Clearance‐Raten und Patientenzufriedenheit.^44^

Die Ergebnisse der vorliegenden Studie sind mit Vorsicht zu interpretieren, da sie auf einer begrenzten Anzahl von Studien mit kleinen Stichprobengrößen beruhen. Zudem wiesen viele der eingeschlossenen Studien ein Verzerrungsrisiko auf, hauptsächlich bedingt durch unzureichende Angaben zur Verblindung und Randomisierung. Zusätzlich können sich laser‐ und energiebasierte Geräte, selbst bei gleicher Wellenlänge, erheblich unterscheiden, und können dadurch sowohl die Wirksamkeit als auch das Sicherheitsprofil der Behandlung wesentlich beeinflussen.

Kombinationstherapien werden häufig für die Behandlung fortgeschrittener Rosazea‐Verläufe empfohlen.^9^ In unserer Analyse wurden alle Studien eingeschlossen, bei denen mindestens ein Behandlungsarm eine Laser‐ oder EBD‐Therapie umfasste. Es lagen jedoch nur wenige Studien vor, die sich explizit mit Kombinationstherapien beschäftigten. Eine individualisierte Behandlung, die sich am jeweiligen klinischen Phänotyp der Rosazea orientiert, ist entscheidend für den therapeutischen Erfolg. Kombinationstherapien, sei es durch den Einsatz mehrerer Geräte oder durch die Kombination von Lasern oder EBDs mit topischen oder systemischen Wirkstoffen, zeigen vielversprechendes Potenzial zur Verbesserung der Patientenergebnisse, insbesondere angesichts der vielschichtigen Symptomatik der Erkrankung.^45^ Weitere Studien sind erforderlich, um die Wirksamkeit und Sicherheit dieser kombinierten Ansätze umfassend zu bewerten.

Patienten und Ärzte sollten sich im Vorfeld über die zu erwartenden Behandlungsergebnisse besprechen, da Laser‐ und EBD‐Therapien in der Regel nur vorübergehende Linderung verschaffen und häufig mehrere Sitzungen für einen nachhaltigen Effekt erforderlich sind. Die Patienten sollten umfassend über den Behandlungsverlauf informiert werden, einschließlich der voraussichtlichen Therapiedauer, des Heilungsprozesses, der zu erwartenden Resultate sowie möglicher Risiken oder Komplikationen, die mit der jeweiligen Laser‐ oder EBD‐Therapie verbunden sein können.

Die eingeschlossenen Studien bezogen sich überwiegend auf weibliche Patienten. Epidemiologische Untersuchungen zeigen jedoch eine gleichmäßige Geschlechterverteilung, was darauf hindeutet, dass der höhere Frauenanteil in den Behandlungsstudien eher auf ein unterschiedliches Inanspruchnahmeverhalten im Gesundheitswesen als auf eine tatsächliche Differenz in der Krankheitsprävalenz zurückzuführen ist.^46^

Hinsichtlich der Hauttypen hatten weniger als 3% der eingeschlossenen Patienten einen Fitzpatrick‐Hauttyp V. Es gab keine Studie, die Patienten mit dem Hauttyp VI einschloss. Dies spiegelt die Beobachtung wider, dass die Rosazea bei Personen mit dunklerer Haut seltener diagnostiziert wird und dass ein höheres Risiko für Nebenwirkungen bei Laserbehandlungen besteht.^47^ Symptome wie ein transientes oder persistierendes Erythem sind bei dunkler Haut oft weniger auffällig, was zur späten Diagnose beitragen kann.^48^ Eine systematische Übersichtsarbeit fand keine Unterschiede in der Prävalenz von Rosazea in Abhängigkeit von geografischer oder ethnischer Herkunft, was unterstreicht, dass die Unterschiede eher auf fehlender Erkennung als auf einer geringeren Häufigkeit beruhen.^1^, ^49^ Dennoch muss beachtet werden, dass viele der bei Rosazea eingesetzten Laser‐ und energiebasierten Therapien für dunklere Hauttypen weniger geeignet sind, da die Melaninabsorption in diesen Hauttypen zu hoch sein kann.

Diese Studie weist einige Limitationen auf. Die eingeschlossenen Studien berücksichtigten weder Hauttyp, Alter noch Schweregrad der Rosazea, Faktoren, die das klinische Ergebnis erheblich beeinflussen könnten. Zudem war die Stichprobengröße in vielen Studien zu klein, um weiterführende Subgruppenanalysen durchführen zu können. Langzeituntersuchungen fehlen ebenfalls, was die Bewertung der Nachhaltigkeit der Behandlungseffekte und möglicher Rezidivraten erschwert. Der Fokus auf RCTs könnte wichtige Erkenntnisse aus nichtrandomisierten Studien ausschließen, wurde jedoch bewusst gewählt, um die Evidenzbasis möglichst hochwertig zu halten.

Zusammenfassend bietet die vorliegende systematische Übersichtsarbeit mit NMA eine umfassende Bewertung von laser‐ und energiebasierten Therapien bei Rosazea auf Grundlage verfügbarer RCTs. Die Ergebnisse sollten jedoch mit Vorsicht interpretiert werden, da die meisten eingeschlossenen Studien ein insgesamt hohes bis unklareres Verzerrungsrisiko aufwiesen. Um die Evidenzlage zu verbessern, sind weitere gut konzipierte Studien erforderlich, die die Wirksamkeit und Sicherheit der einzelnen Verfahren weiterführend untersuchen. Zudem sind Daten zu den langfristigen Auswirkungen laser‐ und energiebasierter Therapien auf den Krankheitsverlauf bislang begrenzt. Wie bei anderen chronischen Erkrankungen liegt das primäre Therapieziel nicht nur in der Heilung, sondern in der wirksamen Symptomkontrolle. Zukünftige Forschung sollte sich auf die Optimierung von Behandlungsparametern, Intervallen, patientenspezifischen Faktoren und Kombinationstherapien konzentrieren, um eine personalisierte und effektivere Therapie zu ermöglichen.

## DANKSAGUNG

Open access Veröffentlichung ermöglicht und organisiert durch Projekt DEAL.

## FINANZIERUNG

Die Studie wurde unterstützt von der Deutschen Dermatologischen Lasergesellschaft (DDL).

## INTERESSENKONFLIKT

L.N. und K.H. erhielten Vortragshonorare von Cynosure Lutronic^®^. Alle anderen Autoren erklären keinen Interessenkonflikt.

## Supporting information



Supplementary information

Supplementary information

Supplementary information

Supplementary information

Supplementary information
