# Supplementary material for: Laser‐ und energiebasierte Systeme zur Behandlung der Rosazea – ein systematischer Review mit Netzwerk‐Metaanalyse
Source: J Dtsch Dermatol Ges. 2026 Jan 14;24(1):24–33. [Article in German] doi: 10.1111/ddg.15961_g (PMC12800870; doi:10.1111/ddg.15961_g)
Supplement: Supplementary file 2 — Supplementary information [file DDG-24-24-s005.docx]

**Charakteristika der eingeschlossenen Studien [geordnet nach Studien-ID]**

| **Alam 2013** | | |
| --- | --- | --- |
| Methode | Prospektive, randomisierte, kontrollierte Split-Face-Studie | |
| Teilnehmende | - Patienten mit Rosacea erythemato-teleangiectatica - Gesamt n = 16; weiblich n = 8; 2 Abbrüche aufgrund posttherapeutischer Schwellung - Mittleres Alter: 42 Jahre - Hauttyp nach Fitzpatrick: I (n = 1); II (n = 11); III (n = 2) | |
| Intervention | A: Nd:YAG-Laser  B: FSL | |
| Endpunkte | - Nachbeobachtung: 4 Wochen nach Behandlung - prozentuale Differenz im Spektralphotometer - bevorzugte behandelte Gesichtshälfte - Behandlungszufriedenheit per Fragebogen - Schmerzintensität | |
| Weiteres | Parameter:   - 4 Sitzungen im Abstand von 3-4 Wochen - 1064 nm Nd:YAG-Laser: 6 J/cm²; 8 mm Spot; 0,3 ms Pulsdauer - FSL: 7,5 J/cm²; 10 mm Spot; 6 ms Pulsdauer; 30 ms/20 ms DCD (Dynamic Cooling Device); 1 Pass mit 15 % Überlappung | |
| **Risk of bias** | | |
| **Bias** | **Urteil der Autoren** | **Begründung für das Urteil** |
| Generierung der Randomisierungssequenz (selection bias) | gering | Zahlengenerator |
| verdeckte Gruppenzuteilung (selection bias) | gering | undurchsichtige, nummerierte Umschläge |
| Verblindung von Teilnehmern und Studienpersonal (performance bias) | gering | Probanden verblindet; erster Prüfer nicht |
| Verblindung der Endpunkterhebung (detection bias) | gering | Prüfer verblindet |
| unvollständige Daten zu Endpunkten (atrition bias) | gering | 2 Ausfälle wegen Nebenwirkungen |
| selektives Berichten zu Endpunkten (reporting bias) | gering | Wir stuften dies als geringes Risiko für Verzerrungen ein. |
| andere Ursachen für Bias | gering | Die Studie schien frei von anderen Formen der Verzerrung zu sein. |

| **Campos 2019** | | |
| --- | --- | --- |
| Methode | Prospektive, randomisierte, kontrollierte, doppelblinde Split-Face-Studie | |
| Teilnehmende | - Rosazea erythemato-teleangiectatica - Gesamt n = 29; weiblich n = 17; 2 Abbrüche wegen Purpura oder Schmerzen während Behandlung - Mittleres Alter: 52,9 Jahre - Hauttyp nach Fitzpatrick: keine Angaben | |
| Intervention | A: FSL  B: FSL + Nd:YAG-Laser | |
| Endpunkte | - Nachbeobachtung: vor jeder Sitzung, 1 Monat nach Abschluss - Spektralphotometer - Schmerz (0–10) - Nebenwirkungen - Purpura (%) - Zufriedenheit (0–100 %) - Weiterempfehlung | |
| Weiteres | - Parameter: 3 Sitzungen im Abstand von 3-4 Wochen - FSL: 6,0 J/cm²; 7 mm Spot; 0,5 ms Pulsdauer; Kühlung Stufe 3/5; 1 Pass mit 10 % Überlappung - FSL + Nd:YAG-Laser: 7,0 J/cm² FSL + 35 J/cm² Nd:YAG-Laser; 7 mm Spot; 10 ms FSL, 15 ms Nd:YAG-Laser; Kühlung Stufe 3/5; 1 Pass mit minimaler Überlappung | |
| **Risk of bias** | | |
| **Bias** | **Urteil der Autoren** | **Begründung für das Urteil** |
| Generierung der Randomisierungssequenz (selection bias) | gering | Zahlengenerator |
| verdeckte Gruppenzuteilung (selection bias) | gering | undurchsichtige, nummerierte Umschläge |
| Verblindung von Teilnehmern und Studienpersonal (performance bias) | gering | Die Probanden waren verblindet. Der erste Prüfer war aufgrund der Art der Behandlung nicht verblindet. |
| Verblindung der Endpunkterhebung (detection bias) | gering | Die Probanden und Prüfer für die spektroskopische Analyse waren verblindet. |
| unvollständige Daten zu Endpunkten (atrition bias) | gering | 2 Probanden brachen die Studie aufgrund von Purpura nach der Behandlung oder Schmerzen während der Behandlung ab. |
| selektives Berichten zu Endpunkten (reporting bias) | gering | Wir stuften dies als geringes Risiko für Verzerrungen ein. |
| andere Ursachen für Bias | gering | Die Studie schien frei von anderen Formen der Verzerrung zu sein. |

| **Handler 2017** | | |
| --- | --- | --- |
| Methode | Prospektive, randomisierte Split-Side-Studie | |
| Teilnehmende | - Patienten mit Gesichtserythem - Gesamt n = 15; weiblich n = 11; 13 bei Tag 30, 14 bei Tag 90 ausgewertet - Mittleres Alter: 53,9 Jahre - Hauttyp nach Fitzpatrick: I–II | |
| Intervention | A: IPL  B: FSL | |
| Endpunkte | - Nachbeobachtung: 90 Tage nach Behandlung - Veränderung des Erythems (5-Punkte-Skala) - Nebenwirkungen | |
| Weiteres | - Parameter: - 2 Sitzungen im Abstand von 30 Tagen - IPL: 20 J/cm²; 30 ms; 20 °C - FSL: 7 J/cm²; 30 ms; 20 °C | |
| **Risk of bias** | | |
| **Bias** | **Urteil der Autoren** | **Begründung für das Urteil** |
| Generierung der Randomisierungssequenz (selection bias) | unklar | Keine Informationen verfügbar. |
| verdeckte Gruppenzuteilung (selection bias) | unklar | Keine Informationen verfügbar. |
| Verblindung von Teilnehmern und Studienpersonal (performance bias) | unklar | Keine Informationen verfügbar. |
| Verblindung der Endpunkterhebung (detection bias) | unklar | Keine Informationen verfügbar. |
| unvollständige Daten zu Endpunkten (atrition bias) | gering | Wir stuften dies als geringes Risiko für Verzerrungen ein. |
| selektives Berichten zu Endpunkten (reporting bias) | gering | Wir stuften dies als geringes Risiko für Verzerrungen ein. |
| andere Ursachen für Bias | gering | Die Studie schien frei von anderen Formen der Verzerrung zu sein. |

| **Iyer 2006** | | |
| --- | --- | --- |
| Methode | Prospektive, randomisierte Split-Side-Studie | |
| Teilnehmende | - Patienten mit Gesichtserythem und Teleangiektasien - Gesamt n = 9; weiblich n = 6. - Mittleres Alter: keine weiteren Angaben - Hauttyp nach Fitzpatrick: I – II; keine weiteren Angaben | |
| Intervention | A: FSL mit purpurischer Reaktion  B: FSL mit subpurpurischer Reaktion | |
| Endpunkte | - Nachbeobachtung: 3 Wochen nach Behandlung - Erythem (4-Punkte-Skala) - Gefäßdurchmesser (4-Punkte-Skala) - Dichte der Teleangiektasien - Gefäßverzweigung (5-Punkte-Skala) | |
| Weiteres | Parameter:   - Purpurisch: 12 J/cm²; 7 mm; 6 ms; DCD 20/10 ms; 1 Pass ohne Überlappung - Subpurpurisch: 6 J/cm²; 10 mm; 6 ms; DCD 20/10 ms; 4 Passe | |
| **Risk of bias** | | |
| **Bias** | **Urteil der Autoren** | **Begründung für das Urteil** |
| Generierung der Randomisierungssequenz (selection bias) | unklar | Keine Informationen verfügbar. |
| verdeckte Gruppenzuteilung (selection bias) | unklar | Keine Informationen verfügbar. |
| Verblindung von Teilnehmern und Studienpersonal (performance bias) | unklar | Keine Informationen verfügbar. |
| Verblindung der Endpunkterhebung (detection bias) | unklar | Keine Informationen verfügbar. |
| unvollständige Daten zu Endpunkten (atrition bias) | hoch | Keine Informationen zu Baseline-Charakteristiken der Patienten verfügbar. |
| selektives Berichten zu Endpunkten (reporting bias) | gering | Wir stuften dies als geringes Risiko für Verzerrungen ein. |
| andere Ursachen für Bias | hoch | Kleine Stichprobe (n = 9). |

| **Karppinen 2019** | | |
| --- | --- | --- |
| Methode | Prospektive, randomisierte, doppelblinde Split-Side-Studie | |
| Teilnehmende | - Symmetrische Teleangiektasien im Gesicht - Gesamt n = 24; weiblich n = 16; 6 ausgeschlossen (nicht auswertbar). - Mittleres Alter: 48 Jahre - Hauttyp anch Fitzpatrick: I (n = 6); II (n = 8); III (n = 4) | |
| Intervention | A: KTP-Laser  B: Yellow-Laser | |
| Endpunkte | - Nachbeobachtung: 48–72 h Nebenwirkungen, 1–2 Monate Effektivität - Teleangiektasien (7-Punkte-Skala) - Schmerz (0–100) - Erythem, Krusten, Purpura, Blasen (4-Punkte-Skala) | |
| Weiteres | Parameter:   - 1-2 Sitzungen im Abstand von 1-2 Monaten - KTP-Laser: 20–30 J/cm²; 1 mm Spot; 10 ms Puls - Yellow-Laser: 5,6-8,1 J/cm²; 1,4 mm Spot; 25 ms Puls | |
| **Risk of bias** | | |
| **Bias** | **Urteil der Autoren** | **Begründung für das Urteil** |
| Generierung der Randomisierungssequenz (selection bias) | gering | Es wurde ein webbasiertes validiertes Programm (Research Randomizer) verwendet. |
| verdeckte Gruppenzuteilung (selection bias) | unklar | Keine Informationen verfügbar. |
| Verblindung von Teilnehmern und Studienpersonal (performance bias) | gering | Die Probanden waren verblindet. Der erste Prüfer war aufgrund der Art der Behandlung nicht verblindet. |
| Verblindung der Endpunkterhebung (detection bias) | unklar | Die Prüfer, die die Behandlungen durchführten, bewerteten auch das Ergebnis. Obwohl die Bewertungsbilder keine Informationen über den verwendeten Laser enthielten, wodurch die Studie verblindet erschien, besteht dennoch das potenzielle Risiko, dass die Prüfer ihre eigenen Patienten erkannt haben. |
| unvollständige Daten zu Endpunkten (atrition bias) | gering | Wir stuften dies als geringes Risiko für Verzerrungen ein. |
| selektives Berichten zu Endpunkten (reporting bias) | gering | Wir stuften dies als geringes Risiko für Verzerrungen ein. |
| andere Ursachen für Bias | gering | Wir stuften dies als geringes Risiko für Verzerrungen ein. |

| **Karsai 2008** | | |
| --- | --- | --- |
| Methode | Prospektive, randomisierte, kontrollierte, einfach verblindete Split-Side-Studie | |
| Teilnehmende | - Patienten mit ausgeprägten Teleangiektasien an der Nase - Gesamt n = 24; weiblich n = 6 - Mittleres Alter: 62,4 ± 12,3 Jahre - Hauttyp nach Fitzpatrick: I–III | |
| Intervention | A: FSL + Nd:YAG-Laser  B: FSL  C: Nd:YAG-Laser | |
| Endpunkte | - Nachbeobachtung: 4 Wochen nach Behandlung - Clearance (4-Punkte-Skala) | |
| Weiteres | Parameter:   - FSL: 595 nm; 10 J/cm²; 7 mm Spot; 10 ms Pulsdauer - Nd:YAG-Laser: 70 J/cm²; 50 ms Pulsdauer; Multiplex-Interpulse-Delay 100 ms - Kühlung: Luftkühlung Stufe 4 während Behandlung, Kühlung mit Eis danach | |
| **Risk of bias** | | |
| **Bias** | **Urteil der Autoren** | **Begründung für das Urteil** |
| Generierung der Randomisierungssequenz (selection bias) | unklar | Keine Informationen verfügbar. |
| verdeckte Gruppenzuteilung (selection bias) | unklar | Keine Informationen verfügbar. |
| Verblindung von Teilnehmern und Studienpersonal (performance bias) | unklar | Keine Informationen verfügbar. |
| Verblindung der Endpunkterhebung (detection bias) | gering | Drei unabhängige Prüfer beurteilten die Endpunkte. |
| unvollständige Daten zu Endpunkten (atrition bias) | gering | Wir stuften dies als geringes Risiko für Verzerrungen ein. |
| selektives Berichten zu Endpunkten (reporting bias) | gering | Wir stuften dies als geringes Risiko für Verzerrungen ein. |
| andere Ursachen für Bias | gering | Wir stuften dies als geringes Risiko für Verzerrungen ein. |

| **Kim 2011** | | |
| --- | --- | --- |
| Methode | Prospektive, randomisierte, offene Split-Side-Studie | |
| Teilnehmende | - Patienten mit Rosazea erythemato-teleangiectatica oder papulo-pustulosa - Gesamt n = 18; weiblich n = 13; 3 Abbrüche (fehlende Nachsorge) - Mittleres Alter: 49 Jahre (29–67) - Hauttyp nach Fitzpatrick: III–V, davon 62 % Typ IV | |
| Intervention | A: FSL + Niacin  B: FSL | |
| Endpunkte | - Nachbeobachtung: 6 Wochen nach Behandlung. - Verbesserung des Erythems (4-Punkte-Skala) - Patientenzufriedenheit (10-Punkte-Skala) | |
| Weiteres | Parameter:   - 3 Sitzungen im Abstand von 3 Wochen - Niacin-Creme: dünn aufgetragen, nach 20 min mit 0,9 % Kochsalz abgewaschen - FSL: 7-9 J/cm²; 7 mm Spot; 10 ms Pulsdauer; 10 % Überlappung; Luftkühlung Stufe 3-4; Eispackungen nach Behandlung | |
| **Risk of bias** | | |
| **Bias** | **Urteil der Autoren** | **Begründung für das Urteil** |
| Generierung der Randomisierungssequenz (selection bias) | unklar | Keine Informationen verfügbar. |
| verdeckte Gruppenzuteilung (selection bias) | hoch | keine Verblindung für Behandler |
| Verblindung von Teilnehmern und Studienpersonal (performance bias) | hoch | Aufgrund der Art des Studiendesigns wurden die Probanden und Behandler nicht verblindet. |
| Verblindung der Endpunkterhebung (detection bias) | gering | Drei verblindete Dermatologen |
| unvollständige Daten zu Endpunkten (atrition bias) | gering | Wir stuften dies als geringes Risiko für Verzerrungen ein. |
| selektives Berichten zu Endpunkten (reporting bias) | gering | Wir stuften dies als geringes Risiko für Verzerrungen ein. |
| andere Ursachen für Bias | gering | Wir stuften dies als geringes Risiko für Verzerrungen ein. |

| **Kim 2017** | | |
| --- | --- | --- |
| Methode | Prospektive, randomisierte, kontrollierte, einfach verblindete Split-Face-Studie | |
| Teilnehmende | - Patienten mit Rosazea erythemato-teleangiectatica oder papulo-pustulosa - Gesamt n = 30; weiblich n = 19 - Mittleres Alter: 43,4 Jahre (35–69) - Hauttyp nach Fitzpatrick: keine Angabe | |
| Intervention | A: Radiofrequenz  B: FSL | |
| Endpunkte | - Nachbeobachtung: Woche 4, 8 und 12; 4 Wochen nach Behandlung - Rosazea-Symptome (u. a. Flush, Erythem, Papeln/Pusteln, Teleangiektasien, Brennen, Plaques, Trockenheit, Ödeme, okulare Symptome, phymatöse Veränderungen) – 4-Punkte-Skala - Chromameter - Therapeutische Verbesserung (4-Punkte-Skala) - Patientenzufriedenheit (4-Punkte-Skala) - Nebenwirkungen und Schmerz (0–10 Skala) | |
| Weiteres | Parameter:   - 3 Sitzungen im Abstand von 4 Wochen - FSL: 8-9 J/cm²; 7 mm Spot; 6 ms Puls; 30 ms Kryogenkühlung 30 ms vor Laserimpuls - Radiofrequenz: 2 cm² Applikator; 80–120 J/cm²; 3 °C Kontaktkühlung; 3 Passes | |
| **Risk of bias** | | |
| **Bias** | **Urteil der Autoren** | **Begründung für das Urteil** |
| Generierung der Randomisierungssequenz (selection bias) | unklar | Keine Informationen verfügbar. |
| verdeckte Gruppenzuteilung (selection bias) | hoch | Aufgrund der Art des Studiendesigns wurde die Randomisierung gegenüber den Patienten und Ärzten, die die Behandlungen durchführten, nicht verdeckt. |
| Verblindung von Teilnehmern und Studienpersonal (performance bias) | hoch | Aufgrund der Art des Studiendesigns gab es keine Verblindung seitens der Patienten und Behandler. |
| Verblindung der Endpunkterhebung (detection bias) | gering | Zwei verblindete Dermatologen bewerteten das Ergebnis. |
| unvollständige Daten zu Endpunkten (atrition bias) | gering | Wir stuften dies als geringes Risiko für Verzerrungen ein. |
| selektives Berichten zu Endpunkten (reporting bias) | gering | Wir stuften dies als geringes Risiko für Verzerrungen ein. |
| andere Ursachen für Bias | gering | Wir stuften dies als geringes Risiko für Verzerrungen ein. |

| **Kim 2018** | | |
| --- | --- | --- |
| Methods | Prospektive, randomisierte, kontrollierte, einfach verblindete Split-Side-Studie | |
| Methode | - Patienten mit Rosazea - Gesamt n = 9; weiblich n = 8 - Mittleres Alter: 40,33 ± 13,13 Jahre (20–59) - Fitzpatrick-Hauttyp: II–IV | |
| Teilnehmende | A: kurz-gepulster IPL  B: FSL | |
| Intervention | - Nachbeobachtung: Woche 3, 6, 9, 12 und 15; 3 Wochen nach Behandlung. - Globalbewertung (Arzt und Patient) - Erythem, Teleangiektasien, Papeln/Pusteln, Zufriedenheit (5-Punkte-Skala) - Erythem- und Melaninindex (Spektralphotometrie) - Nebenwirkungen - Schmerz (0–10 Skala) | |
| Endpunkte | Parameter:   - 4 Sitzungen im Abstand von 3 Wochen - IPL: 555 nm Cut-off; 8 J/cm²; 1,5 ms Puls - FSL: 8 J/cm²; 7 mm Spot; 1,5 ms Puls - Kühlung mit Eis nach Behandlung | |
| Weiteres | | |
| **Risk of bias** | **Urteil der Autoren** | **Begründung für das Urteil** |
| **Bias** | unklar | Keine Informationen verfügbar. |
| Generierung der Randomisierungssequenz (selection bias) | unklar | Keine Informationen verfügbar. |
| verdeckte Gruppenzuteilung (selection bias) | unklar | Keine Informationen verfügbar. |
| Verblindung von Teilnehmern und Studienpersonal (performance bias) | gering | Verblindete Dermatologen beurteilten das Ergebnis. |
| Verblindung der Endpunkterhebung (detection bias) | gering | Wir stuften dies als geringes Risiko für Verzerrungen ein. |
| unvollständige Daten zu Endpunkten (atrition bias) | gering | Wir stuften dies als geringes Risiko für Verzerrungen ein. |
| selektives Berichten zu Endpunkten (reporting bias) | hoch | Kleine Stichprobe (n = 9). Es wurden keine Informationen zu Interessenkonflikten vorgelegt. |

| **Luo 2020** | | |
| --- | --- | --- |
| Methode | Prospektive, randomisierte, kontrollierte Split-Side-Studie | |
| Teilnehmende | - Patienten mit Rosacea papulo-pustulosa - Gesamt n = 260; weiblich n = 214; in der IPL-Gruppe 23 Abbrüche (17 Lost to Follow-up, 6 Nebenwirkungen), in der Kontrollgruppe 10 Abbrüche (6 Lost to Follow-up, 4 Verschlechterung der Hautläsionen). - Mittleres Alter: 40,20 ± 10,76 Jahre - Hauttyp nach Fitzpatrick: III–IV | |
| Intervention | A: IPL  B: keine Behandlung | |
| Endpunkte | - Nachbeobachtung: monatlich in den ersten 6 Monaten, danach halbjährlich über 1,5 Jahre - Teleangiektasien (4-Punkte-Skala) - Gesamteffektivität (Symptome wie Flush, Erythem, Teleangiektasien, Brennen, Plaques, Trockenheit, Ödeme, globale Bewertung durch Arzt und Patient) - Rückfallrate | |
| Weiteres | Parameter:   - Nach Milbenbehandlung: 3 Sitzungen im Abstand von 4 Wochen - 540 nm IPL: 10–16 J/cm²; Spot 1,5 × 4 cm²; 12 ms Puls; 10–15 ms Pulsintervall; keine Überlappung - Kühlung mit Eis nach Behandlung | |
| **Risk of bias** | | |
| **Bias** | **Urteil der Autoren** | **Begründung für das Urteil** |
| Generierung der Randomisierungssequenz (selection bias) | unklar | Keine Informationen verfügbar. |
| verdeckte Gruppenzuteilung (selection bias) | unklar | Keine Informationen verfügbar. |
| Verblindung von Teilnehmern und Studienpersonal (performance bias) | hoch | Aufgrund der Art des Studiendesigns wurde keine Verblindung durchgeführt. |
| Verblindung der Endpunkterhebung (detection bias) | unklar | Keine Informationen verfügbar. |
| unvollständige Daten zu Endpunkten (atrition bias) | gering | Wir stuften dies als geringes Risiko für Verzerrungen ein. |
| selektives Berichten zu Endpunkten (reporting bias) | gering | Wir stuften dies als geringes Risiko für Verzerrungen ein. |
| andere Ursachen für Bias | gering | Wir stuften dies als geringes Risiko für Verzerrungen ein. |

| **Maxwell 2010** | | |
| --- | --- | --- |
| Methode | Prospektive, randomisierte, kontrollierte Split-Side-Studie | |
| Teilnehmende | - Patienten mit Rosazea Typ I - Gesamt n = 14; weiblich n = 10; 2 Abbrüche wegen Verschlechterung der Hautläsionen. - Mittleres Alter: 44 Jahre (31–71) - Hauttyp nach Fitzpatrick: keine Angabe | |
| Intervention | A: KTP-Laser  B: keine Behandlung | |
| Endpunkte | - Nachbeobachtung: insgesamt 8 Visiten; 2 Wochen nach Behandlung - Verbesserung von Erythem und Teleangiektasien (5-Punkte-Skala) | |
| Weiteres | Parameter:   - 6 Sitzungen im Abstand von 2 Wochen - 532 nm KTP-Laser: 17-20 J/cm²; 700 µm Spot; 25-30 ms Pulsdauer | |
| **Risk of bias** | | |
| **Bias** | **Urteil der Autoren** | **Begründung für das Urteil** |
| Generierung der Randomisierungssequenz (selection bias) | niedrig | Gewürfelt. |
| verdeckte Gruppenzuteilung (selection bias) | unklar | Keine Informationen verfügbar. |
| Verblindung von Teilnehmern und Studienpersonal (performance bias) | hoch | Aufgrund der Art des Studiendesigns wurde keine Verblindung durchgeführt. |
| Verblindung der Endpunkterhebung (detection bias) | niedrig | Prüfer waren verblindet. |
| unvollständige Daten zu Endpunkten (atrition bias) | hoch | Details über die statistische Analyse fehlen. |
| selektives Berichten zu Endpunkten (reporting bias) | niedrig | Wir stuften dies als geringes Risiko für Verzerrungen ein. |
| andere Ursachen für Bias | hoch | Kleine Stichprobe (n = 14). |

| **Nam 2019** | | |
| --- | --- | --- |
| Methode | Prospektive, randomisierte, kontrollierte Split-Face-Studie | |
| Teilnehmende | - Patienten mit diffusem Gesichtserythem und Teleangiektasien - Gesamt n = 20; weiblich n = 15; 1 Abbruch (persönliche Gründe) - Mittleres Alter: 41,5 Jahre (21–59) - Hauttyp für Fitzpatrick: keine Angabe | |
| Intervention | A: Dioden-gepulster, fraktionierter KTP-Laser  B: FSL | |
| Endpunkte | - Nachbeobachtung: 4 Wochen nach Behandlung - Teleangiektasien (10-Punkte-Skala) - Erythem (Kolorimetrie) - Selbstbewertung des Patienten (6-Punkte-Skala) - Zufriedenheit (6-Punkte-Skala) - Schmerzintensität - Nebenwirkungen | |
| Weiteres | Parameters:   - 3 Sitzungen im Abstand von 4 Wochen - KTP: 0,035 J/cm² Mikrofluenz; 3,86 J/cm² Gesamtfluenz; 20 × 20 mm² Spot; 5 ms Pulsdauer; 3 Passes; keine Überlappung - FSL: 7,5 J/cm²; 10 mm Spot; 6 ms Pulsdauer; 1 Pass; 15 % Überlappung - Kühlung mit Eis nach Behandlung | |
| **Risk of bias** | | |
| **Bias** | **Urteil der Autoren** | **Begründung für das Urteil** |
| Generierung der Randomisierungssequenz (selection bias) | unklar | Keine Informationen verfügbar. |
| verdeckte Gruppenzuteilung (selection bias) | unklar | Keine Informationen verfügbar. |
| Verblindung von Teilnehmern und Studienpersonal (performance bias) | unklar | Keine Informationen verfügbar. |
| Verblindung der Endpunkterhebung (detection bias) | niedrig | Zwei unabhängige Dermatologen bewerteten das Ergebnis. Den seriellen klinischen Fotos wurden zufällige Codes zugewiesen. |
| unvollständige Daten zu Endpunkten (atrition bias) | niedrig | Wir stuften dies als geringes Risiko für Verzerrungen ein. |
| selektives Berichten zu Endpunkten (reporting bias) | niedrig | Wir stuften dies als geringes Risiko für Verzerrungen ein. |
| andere Ursachen für Bias | niedrig | Wir stuften dies als geringes Risiko für Verzerrungen ein. |

| **Neuhaus 2009** | | |
| --- | --- | --- |
| Methode | Prospektive, randomisierte, kontrollierte, einfach verblindete Split-Side-Studie | |
| Teilnehmende | - Patienten mit moderater Rosacea erythemato-teleangiectatica - Gesamt n = 29; weiblich n = 20; 1 Abbruch wegen Schwellung/Reaktion - Mittleres Alter: 45,7 ± 10,6 Jahre - Hauttyp nach Fitzpatrick: I (n = 8); II (n = 19); III (n = 2) | |
| Intervention | A: nicht-purpuragener FSL  B: IPL  C: keine Behandlung | |
| Endpunkte | - Nachbeobachtung: bei jeder Visite, 4 Wochen nach Behandlung - Reflektions-Spektralphotometer - Erythem und Teleangiektasien (4-Punkte-Skala) - Patienteneinschätzung (VAS) für verschiedene Symptome und Gesamtverbesserung - Bereitschaft zur Wiederholung der Behandlung | |
| Weiteres | Parameter:   - 3 Sitzungen im Abstand von 4 Wochen - IPL: 560 nm; 25 J/cm²; 2,4 und 6,0 ms mit 15 ms Abstand - FSL: 7 J/cm²; 10 mm Spot; 6 ms Pulsdauer | |
| **Risk of bias** | | |
| **Bias** | **Urteil der Autoren** | **Begründung für das Urteil** |
| Generierung der Randomisierungssequenz (selection bias) | niedrig | Zahlengenerator. |
| verdeckte Gruppenzuteilung (selection bias) | hoch | Keine Verblindung. |
| Verblindung von Teilnehmern und Studienpersonal (performance bias) | hoch | Die Patienten und der Behandler waren über die Zuordnung informiert. |
| Verblindung der Endpunkterhebung (detection bias) | niedrig | Ein verblindeter Prüfer bewertete das Ergebnis. |
| unvollständige Daten zu Endpunkten (atrition bias) | hoch | Es wurden p-Werte angegeben, aber keine weiteren Details. |
| selektives Berichten zu Endpunkten (reporting bias) | niedrig | Wir stuften dies als geringes Risiko für Verzerrungen ein. |
| andere Ursachen für Bias | niedrig | Wir stuften dies als geringes Risiko für Verzerrungen ein. |

| **Nymann 2010** | | |
| --- | --- | --- |
| Methode | Prospektive, randomisierte, kontrollierte Split-Side-Studie | |
| Teilnehmende | - Patienten mit Rosazea-assoziierten Teleangiektasien - Gesamt n = 40; Geschlechterverteilung nicht angegeben; 1 Todesfall (nicht behandlungsbedingt) - Mittleres Alter: 54 Jahre (42-63) - Hauttyp nach Fitzpatrick: I (n = 1); II (n = 19); III (n = 19) | |
| Intervention | A: FSL  B: IPL | |
| Endpunkte | - Nachbeobachtung: 3 Monate nach Behandlung. - Teleangiektasien (5-Punkte-Skala) - Schmerz (0–10 Skala) - Nebenwirkungen | |
| Weiteres | Parameter:   - 3 Sitzungen im Abstand von 6 Wochen - IPL:   - dünne, oberflächliche Gefäße: PR applicator; 8-16 J/cm^2^; 10 ms; 1 Pass  - dünne, tiefere Gefäße: VL-2 applicator; 10-18 J/cm^2^; 10 ms; 1 Pass  - mittelgroße, oberflächliche Gefäße: 9-15 J/cm^2^; 14 ms; 1 Pass  - mittelgroße, tiefere Gefäße: VL-2 applicator; 11-18 J/cm^2^; 14 ms; 1 Pass  - dicke Gefäße: VL-2 applicator; 12-20 J/cm^2^; 20 ms; 1 Pass   - FSL:   - dünne, oberflächliche Gefäße: 7-9 J/cm^2^; 7 mm; 6 ms; 2-3 pass; DCD 30/10  - dünne, tiefere Gefäße: 6-7,5 J/cm^2^; 10 mm; 6 ms; 2-3 pass; DCD 30/10  - mittelgroße, oberflächliche Gefäße: 8-10 J/cm^2^; 7 mm; 10 ms; 2-3 pass; DCD 30/10  - mittelgroße, tiefere Gefäße: 7-9 J/cm^2^; 10 mm; 6 ms; 2-3 pass; DCD 30/10  - dicke Gefäße: 10-12 J/cm^2^; 7 mm; 20 ms; 2-3 pass; DCD 30/10 | |
| **Risk of bias** | | |
| **Bias** | **Urteil der Autoren** | **Begründung für das Urteil** |
| Generierung der Randomisierungssequenz (selection bias) | niedrig | Die Patienten zogen Lose. |
| verdeckte Gruppenzuteilung (selection bias) | niedrig | Die randomisierte Zuordnung wurde in undurchsichtigen, versiegelten Umschlägen verborgen. |
| Verblindung von Teilnehmern und Studienpersonal (performance bias) | unklar | Die Randomisierung wurde unter Verwendung undurchsichtiger Umschläge durchgeführt. Es wurden jedoch keine weiteren Informationen zur Verblindung der Patienten oder der Prüfer bereitgestellt. |
| Verblindung der Endpunkterhebung (detection bias) | niedrig | Ein verblindeter Prüfer bewertete das Ergebnis. |
| unvollständige Daten zu Endpunkten (atrition bias) | niedrig | Wir stuften dies als geringes Risiko für Verzerrungen ein. |
| selektives Berichten zu Endpunkten (reporting bias) | niedrig | Wir stuften dies als geringes Risiko für Verzerrungen ein. |
| andere Ursachen für Bias | niedrig | Wir stuften dies als geringes Risiko für Verzerrungen ein. |

| **Osman 2022** | | |
| --- | --- | --- |
| Methode | Prospektive, randomisierte, kontrollierte Split-Side-Studie | |
| Teilnehmende | - Patienten mit Rosacea erythemato-teleangiectatica or papulo-pustulosa - Gesamt n = 30; weiblich n = 25 - Mittleres Alter: 38,97 Jahre (16–73) - Hauttyp nach Fitzpatrick: III (n = 26); IV (n = 4) | |
| Intervention | A: FSL + Ivermectin  B: FSL | |
| Endpunkte | - Nachbeobachtung: vor jeder Sitzung, 3 Monate nach Behandlung - Verbesserung (4-Punkte-Skala) - Schweregrad (mild, moderat, schwer) - Zufriedenheit (4-Punkte-Skala) - Dermatoskopie | |
| Weiteres | Parameter:   - 4 Sitzungen im Abstand von 4 Wochen - FSL: 5–6,5 J/cm²; 5–7 mm Spot; 0,45 ms Puls; 1 mm Überlappung - Ivermectin 1 % Creme: 1× täglich, dünn, für 12 Wochen | |
| **Risk of bias** | | |
| **Bias** | **Urteil der Autoren** | **Begründung für das Urteil** |
| Generierung der Randomisierungssequenz (selection bias) | unklar | Keine Informationen verfügbar. |
| verdeckte Gruppenzuteilung (selection bias) | nklar | Keine Informationen verfügbar. |
| Verblindung von Teilnehmern und Studienpersonal (performance bias) | hoch | Aufgrund der Art des Studiendesigns (ohne Verwendung eines Vehikels als Kontrolle). |
| Verblindung der Endpunkterhebung (detection bias) | niedrig | Zwei verblindete unabhängige Prüfer bewerteten das Ergebnis. |
| unvollständige Daten zu Endpunkten (atrition bias) | hoch | p-Werte wurden angegeben, weitere Details wurden jedoch nur teilweise nicht angegeben. |
| selektives Berichten zu Endpunkten (reporting bias) | niedrig | Wir stuften dies als geringes Risiko für Verzerrungen ein. |
| andere Ursachen für Bias | niedrig | Wir stuften dies als geringes Risiko für Verzerrungen ein. |

| **Park 2016** | | |
| --- | --- | --- |
| Methode | Prospektive, randomisierte Split-Side-Studie | |
| Teilnehmende | - Patienten mit milder bis moderater Rosazea - Gesamt n = 21; weiblich n = 20 - Mittleres Alter: 42,9 ± 10,3 Jahre - Hauttyp nach Fitzpatrick: III oder IV | |
| Intervention | A: fraktionierte Mikronadel-Radiofrequenz (FMR)  B: keine Behandlung | |
| Endpunkte | - Nachbeobachtung: bei jeder Sitzung, Woche 4 und 8 nach Behandlung. - Erythema-Index (Dermospectrometer) - a*-Wert (Spektralphotometer) - Histologische Analyse - Schmerz (0–10 Skala) - Zufriedenheit (0–10 Skala) | |
| Weiteres | Parameter:   - 2 Sitzungen im Abstand von 4 Wochen - FMR: 49 isolierte Nadeln; 5-7,5 Watt; 50-70 ms; 2 Passes; leichte Überlappung | |
| **Risk of bias** | | |
| **Bias** | **Urteil der Autoren** | **Begründung für das Urteil** |
| Generierung der Randomisierungssequenz (selection bias) | unklar | Keine Informationen verfügbar. |
| verdeckte Gruppenzuteilung (selection bias) | unklar | Keine Informationen verfügbar. |
| Verblindung von Teilnehmern und Studienpersonal (performance bias) | hoch | Aufgrund der Art des Studiendesigns konnte keine Verblindung der Teilnehmer oder des Personals erreicht werden. |
| Verblindung der Endpunkterhebung (detection bias) | niedrig | Zwei verblindete unabhängige Prüfer bewerteten das Ergebnis. |
| unvollständige Daten zu Endpunkten (atrition bias) | niedrig | Wir stuften dies als geringes Risiko für Verzerrungen ein. |
| selektives Berichten zu Endpunkten (reporting bias) | niedrig | Wir stuften dies als geringes Risiko für Verzerrungen ein. |
| andere Ursachen für Bias | niedrig | Wir stuften dies als geringes Risiko für Verzerrungen ein. |

| **Park 2022** | | |
| --- | --- | --- |
| Methode | Prospektive, randomisierte, einfach verblindete Split-Side-Studie | |
| Teilnehmende | - Patienten mit Rosazea erythemato-teleangiectatica oder papulo-pustulosa - Gesamt n = 27; weiblich n = 18/23; 4 Abbrüche (persönliche Gründe) - Mittleres Alter: 41,5 Jahre (21–64) - Hauttyp für Fitzpatrick: keine Angabe | |
| Intervention | A: Alexandrit-Laser + Nd:YAG-Laser  B: FSL | |
| Endpunkte | - Nachbeobachtung: 1 und 3 Monate nach Behandlung - Erythema-Index (Spektralphotometer) - Globale ästhetische Verbesserung (Skala) - Zufriedenheit (5-Punkte-Skala) - Nebenwirkungen | |
| Weiteres | Parameter:   - 4 Sitzungen im Abstand von 4 Wochen - Alexandrit-Laser: 755 nm; 10 J/cm²; 10 mm Spot; 20 ms Pulsdauer - Nd:YAG-Laser: 1064 nm; 4 J/cm²; 10 mm Spot; 0,3 ms Pulsdauer - FSL: 8 J/cm²; 10 mm Spot; 10 ms Puls; subpurpurisch | |
| **Risk of bias** | | |
| **Bias** | **Urteil der Autoren** | **Begründung für das Urteil** |
| Generierung der Randomisierungssequenz (selection bias) | unklar | Keine Informationen verfügbar. |
| verdeckte Gruppenzuteilung (selection bias) | unklar | Keine Informationen verfügbar. |
| Verblindung von Teilnehmern und Studienpersonal (performance bias) | hoch | Aufgrund der Art des Studiendesigns konnte keine Verblindung der Teilnehmer oder des Personals erreicht werden. |
| Verblindung der Endpunkterhebung (detection bias) | niedrig | Zwei verblindete Prüfer bewerteten das Ergebnis. |
| unvollständige Daten zu Endpunkten (atrition bias) | niedrig | Wir stuften dies als geringes Risiko für Verzerrungen ein. |
| selektives Berichten zu Endpunkten (reporting bias) | niedrig | Wir stuften dies als geringes Risiko für Verzerrungen ein. |
| andere Ursachen für Bias | unklar | Es wurde keine Erklärung zu Interessenkonflikten vorgelegt. |

| **Seo 2013** | | |
| --- | --- | --- |
| Methode | Prospektive, randomisierte, vergleichende, einfach verblindete Parallelgruppen-Studie | |
| Teilnehmende | - Patienten mit Rosacea erythemato-teleangiectatica oder papulo-pustulosa - Gesamt n = 49; weiblich n = 18; 12 Abbrüche (11 Lost-to Follow-up, 1 Verschlechterung nach FSL) - Mittleres Alter: LPAN-Gruppe 49,9 ± 12,8 Jahre; FSL-Gruppe 48,6 ± 11,3 Jahre - Hauttyp nach Fitzpatrick: III (n = 2); IV (n = 30); V (n = 5) | |
| Intervention | A: Langgepulster Alexandrit + Nd:YAG-Laser (LPAN)  B: FSL | |
| Endpunkte | - Nachbeobachtung: 2 Wochen und 6 Monate nach Behandlung. - Erythema-Index (Spektralphotometer) - Ärztliche Gesamtbewertung (4-Punkte-Skala) - Patientenzufriedenheit (5-Punkte-Skala) - Schmerz (0–10 Skala) - Nebenwirkungen | |
| Weiteres | Parameter:   - 4 Sitzungen im Abstand von 4 Wochen - LPAN: Alexandrit 755 nm, 30 J/cm², 12 ms Puls, 10 mm Spot, 2 Passes, 15 % Überlappung - Nd:YAG-Laser: 1064 nm, 3,0 J/cm², 0,5 ms Puls, 10 mm Spot, 6 Passes - FSL: 7 J/cm², 6 ms Puls, 10 mm Spot, 2 Passes, 15 % Überlappung | |
| **Risk of bias** | | |
| **Bias** | **Urteil der Autoren** | **Begründung für das Urteil** |
| Generierung der Randomisierungssequenz (selection bias) | unklar | Jede Probandennummer wurde einem Laser zugewiesen, bevor einem Probanden eine Probandennummer zugewiesen wurde. Da keine weiteren Informationen über den Zugriff des Prüfers auf die Probandennummern vorlagen, haben wir dies als unklares Risiko eingestuft. |
| verdeckte Gruppenzuteilung (selection bias) | unklar | Keine Informationen verfügbar. |
| Verblindung von Teilnehmern und Studienpersonal (performance bias) | hoch | Aufgrund der Art des Studiendesigns wurden die Probanden und Prüfer nicht verblindet. |
| Verblindung der Endpunkterhebung (detection bias) | niedrig | Zwei verblindete Dermatologen bewerteten das Ergebnis. |
| unvollständige Daten zu Endpunkten (atrition bias) | niedrig | Wir stuften dies als geringes Risiko für Verzerrungen ein. |
| selektives Berichten zu Endpunkten (reporting bias) | niedrig | Wir stuften dies als geringes Risiko für Verzerrungen ein. |
| andere Ursachen für Bias | niedrig | Es wurde keine Erklärung zu Interessenkonflikten vorgelegt. |

| **Sodha 2021** | | |
| --- | --- | --- |
| Methode | Prospektive, randomisierte, kontrollierte Parallelgruppen-Studie | |
| Teilnehmende | - Patienten mit Rosacea erythemato-teleangiectatica - Gesamt n = 34; weiblich n = 23; 4 Abbrüche (verschiedene Gründe) - Mittleres Alter: 45 ± 11 Jahre (26–61) - Hauttyp nach Fitzpatrick-: I (n = 1); II (n = 19); III (n = 8); IV (n = 2) | |
| Intervention | A: Oxymethazolin + FSL  B: Oxymetazolin | |
| Endpunkte | - Nachbeobachtung: bei jeder Sitzung, 1 Woche nach erster Sitzung; 3 Monate nach letzter Sitzung. - Ärztliche Erythembewertung (5-Punkte-Skala) - Selbstbewertung (5-Punkte-Skala) - Globale ästhetische Verbesserung (5-Punkte-Skala) - Verbesserung der Gefäßgröße (5-Punkte-Skala) - Zufriedenheit (5-Punkte-Skala) - Schmerz (0–10 Skala) - Nebenwirkungen | |
| Weiteres | Parameter:   - FSL: 3 Sitzungen im Abstand von 1 Monat; Parameter variabel; klinischer Endpunkt: sofortige Gefäßblanchierung oder leichte Blaufärbung mit nachfolgendem Erythem (nicht-purpurisch) - Kühlung: Kontakt oder DCD 30/20 ms Spray / 20 ms Verzögerung - Oxymetazolin 1 % Creme: 1× täglich für 6 Monate | |
| **Risk of bias** | | |
| **Bias** | **Urteil der Autoren** | **Begründung für das Urteil** |
| Generierung der Randomisierungssequenz (selection bias) | unklar | Keine Informationen verfügbar. |
| verdeckte Gruppenzuteilung (selection bias) | unklar | Keine Informationen verfügbar. |
| Verblindung von Teilnehmern und Studienpersonal (performance bias) | hoch | Aufgrund der Art des Studiendesigns wurden die Probanden und Prüfer nicht verblindet. |
| Verblindung der Endpunkterhebung (detection bias) | unklar | Keine Informationen verfügbar. |
| unvollständige Daten zu Endpunkten (atrition bias) | niedrig | Wir stuften dies als geringes Risiko für Verzerrungen ein. |
| selektives Berichten zu Endpunkten (reporting bias) | niedrig | Wir stuften dies als geringes Risiko für Verzerrungen ein. |
| andere Ursachen für Bias | hoch | Ein Autor ist Mitglied des medizinischen Beirats des Laserunternehmens, dessen Lasersystem in der Studie verwendet wurde. |

| **Tanghetti 2011** | | |
| --- | --- | --- |
| Methode | Prospektive, randomisierte, kontrollierte Split-Side-Studie | |
| Teilnehmende | - Patienten mit Gesichtsteleangiektasien - Gesamt n = 16; weiblich n = 14 - Mittleres Alter: 63 Jahre (35–85) - Hauttyp nach Fitzpatrick: keine Angabe | |
| Intervention | A: FSL  B: IPL | |
| Endpunkte | - Nachbeobachtung: 48–96 Stunden und 1–2 Monate (Sicherheit); 3 Monate (Wirksamkeit). - Hautreaktion (4-Punkte-Skala) - Teleangiektasie-Score (TGS, 7-Punkte-Skala) - Zufriedenheit - Empfehlung an Freunde/Familie | |
| Weiteres | Parameter:   - 1–2 Sitzungen im Abstand von 1–2 Monaten - FSL: kleinere Gefäße 8,1-8,5 J/cm², 10 ms, 10 mm Spot; größere Gefäße 14,5 J/cm², 40 ms, 7 mm Spot - FSL für größere Gefäße: 14.5 J/cm^2^; 40 ms; 7 mm Spot; Luftkühlung Stufe 4; Ultraschallgel - IPL für kleinere Gefäße: 34-40 J/cm²; 10 ms; Spotgröße 10 x 15 mm; 2-3 Passes; kein Impulsstapelung; Kontaktkühlung 5 °C; optische Kopplungslotion - IPL für größere Gefäße: 55-70 J/cm²; 100 ms Impulsdauer; 10 x 15 mm Spotgröße; 2–3 Passes; kein Impulsstapeln; 5 °C Kontaktkühlung; optische Kopplungslotion - Klinischer Endpunkt für beide Systeme: Verschwinden der Gefäße oder dunkelgraue bis schwarze Verfärbung der Gefäße | |
| **Risk of bias** | | |
| **Bias** | **Urteil der Autoren** | **Begründung für das Urteil** |
| Generierung der Randomisierungssequenz (selection bias) | niedrig | Münzwurf. |
| verdeckte Gruppenzuteilung (selection bias) | unklar | Keine Informationen verfügbar. |
| Verblindung von Teilnehmern und Studienpersonal (performance bias) | unklar | Keine Informationen verfügbar. |
| Verblindung der Endpunkterhebung (detection bias) | niedrig | Drei verblindete Prüfer bewerteten das Ergebnis. |
| unvollständige Daten zu Endpunkten (atrition bias) | niedrig | Wir stuften dies als geringes Risiko für Verzerrungen ein. |
| selektives Berichten zu Endpunkten (reporting bias) | niedrig | Wir stuften dies als geringes Risiko für Verzerrungen ein. |
| andere Ursachen für Bias | hoch | Die Studie wurde vom Hersteller des IPL-Systems gesponsert. |

| **Tierney 2009** | | |
| --- | --- | --- |
| Methode | Prospektive, randomisierte, kontrollierte Split-Side-Studie | |
| Teilnehmende | - Patienten mit Teleangiektasien im Gesicht - Gesamt n = 10; weiblich n = 6 - Mittleres Alter: 56,4 ± 13,06 Jahre (42–82) - Hauttyp nach Fitzpatrick: keine Angabe | |
| Intervention | A: 532 nm Diodenlaser  B: 940 nm Diodenlaser | |
| Endpunkte | - Nachbeobachtung: bei jeder Sitzung, 2 Monate nach Behandlung - Teleangiektasien (10-Punkte-Skala) - Nebenwirkungen (6-Punkte-Skala) | |
| Weiteres | Parameter:   - 2 Sitzungen im Abstand von 6 Wochen - 532 nm: 15 J/cm²; 60 ms Pulsdauer; 1 mm Spot - 940 nm: 100 J/cm²; 21 ms Pulsdauer; 1 mm Spot - Kühlung: Eis für 5 Minuten | |
| **Risk of bias** | | |
| **Bias** | **Urteil der Autoren** | **Begründung für das Urteil** |
| Generierung der Randomisierungssequenz (selection bias) | niedrig | Münzwurf. |
| verdeckte Gruppenzuteilung (selection bias) | unklar | Keine Informationen verfügbar. |
| Verblindung von Teilnehmern und Studienpersonal (performance bias) | unklar | Keine Informationen verfügbar. |
| Verblindung der Endpunkterhebung (detection bias) | niedrig | Zwei verblindete Prüfer bewerteten das Ergebnis. |
| unvollständige Daten zu Endpunkten (atrition bias) | niedrig | Wir stuften dies als geringes Risiko für Verzerrungen ein. |
| selektives Berichten zu Endpunkten (reporting bias) | niedrig | Wir stuften dies als geringes Risiko für Verzerrungen ein. |
| andere Ursachen für Bias | hoch | Kleine Stichprobe (n = 10). Es wurde keine Erklärung zu Interessenkonflikten vorgelegt. |

| **Uebelhoer 2007** | | |
| --- | --- | --- |
| Methode | Prospektive, randomisierte, einfach verblindete Split-Side-Studie | |
| Teilnehmende | - Patienten mit diffusen Teleangiektasien im Gesicht (Photoaging oder Rosazea) - Gesamt n = 15; weiblich n = 7 - Mittleres Alter: 52,4 Jahre (35–70) - Fitzpatrick-Hauttyp: I (n = 6); II (n = 6); III (n = 2); IV (n = 1) | |
| Intervention | A: KTP-Laser  B: FSL | |
| Endpunkte | - Nachbeobachtung: bei jeder Sitzung, 3 Wochen nach Behandlung. - Patient wählte Seite mit bester Verbesserung - Patient wählte angenehmste Behandlung - Ausmaß posttherapeutischer Nebenwirkungen (Rötung, Schwellung, Krusten) | |
| Weiteres | Parameter:   - 3 Sitzungen im Abstand von 3 Wochen - FSL: 7,5 J/cm²; 10 ms; 10 mm Spot; 1 Pass; ≥ 13 % Überlappung; ggf. 2 Passes bei ausgeprägtem Erythem - KTP-LAser: für größte Gefäße 8-11 J/cm²; 18-20 ms; 5 mm Spot; für gesamten Bereich 7-10 J/cm²; 20–25 ms; 10 mm Spot - Nachbehandlung: Prednison 3 Tage oder Kühlung mit Eis | |
| **Risk of bias** | | |
| **Bias** | **Urteil der Autoren** | **Begründung für das Urteil** |
| Generierung der Randomisierungssequenz (selection bias) | unklar | Jede Probandennummer wurde einem Laser zugewiesen, bevor einem Probanden eine Probandennummer zugewiesen wurde. Da keine weiteren Informationen über den Zugriff des Prüfers auf die Probandennummern vorlagen, haben wir dies als unklares Risiko eingestuft. |
| verdeckte Gruppenzuteilung (selection bias) | unklar | Keine Informationen verfügbar. |
| Verblindung von Teilnehmern und Studienpersonal (performance bias) | hoch | Die Patienten und der die Behandlung durchführende Prüfer waren nicht verblindet. |
| Verblindung der Endpunkterhebung (detection bias) | niedrig | Ein verblindeter Prüfer bewertete das Ergebnis. |
| unvollständige Daten zu Endpunkten (atrition bias) | hoch | Standardabweichungen oder -bereiche und statistische Tests wurden nicht angegeben. |
| selektives Berichten zu Endpunkten (reporting bias) | niedrig | Wir stuften dies als geringes Risiko für Verzerrungen ein. |
| andere Ursachen für Bias | hoch | Die Studie wurde von dem Laserhersteller des KTP-Lasers finanziert. |

| **Üstüner 2008** | | |
| --- | --- | --- |
| Methode | Prospektive, randomisierte, kontrollierte, doppelblinde Split-Side-Studie. | |
| Teilnehmende | - Patienten mit Rosacea erythemato-teleangiectatica und papulo-pustulosa - Gesamt n = 30; weiblich n = 13; 2 Abbrüche wegen Nebenwirkungen - Mittleres Alter: 38,64 ± 13,33 Jahre (22–67) - Hauttyp nach Fitzpatrick: II (n = 19); III (n = 9) | |
| Intervention | A: KTP-Laser  B: Nd:YAG-Laser | |
| Endpunkte | - Nachbeobachtung: vor und nach jeder Sitzung; 6 Monate nach Behandlung - Erythem (4-Punkte-Skala) - Teleangiektasie (4-Punkte-Skala) - Gefäßdicke (4 Kategorien) - Veränderung der Gefäßdicke - Rosazea-Schweregrad (4-Punkte-Skala) - Klinische Verbesserung (4-Punkte-Skala) - Zufriedenheit (4-Punkte-Skala) - Schmerz (0–10 Skala) - Nebenwirkungen - Dermatologischer Lebensqualitätsindex (türkische Version) | |
| Weiteres | Parameter:   - 4 Sitzungen im Abstand von 1 Monat - KTP-Laser: 1,5-2 J/cm²; 3 mm Spot; Luftkühlung Stufe 4; Endpunkt: bläulich-rote Gefäßverfärbung - Nd:YAG-Laser: 90-110 J/cm²; 15 ms Pulsdauer; 4 mm Spot; 5 % Überlappung; Luftkühlung Stufe 4; Endpunkt: komplettes Verschwinden der Gefäße | |
| **Risk of bias** | | |
| **Bias** | **Urteil der Autoren** | **Begründung für das Urteil** |
| Generierung der Randomisierungssequenz (selection bias) | unklar | Keine Informationen verfügbar. |
| verdeckte Gruppenzuteilung (selection bias) | unklar | Keine Informationen verfügbar. |
| Verblindung von Teilnehmern und Studienpersonal (performance bias) | niedrig | Die Studie war doppelt verblindet. |
| Verblindung der Endpunkterhebung (detection bias) | niedrig | Die Studie war doppelt verblindet. |
| unvollständige Daten zu Endpunkten (atrition bias) | hoch | p-Werte wurden angegeben, weitere Details wurden jedoch teilweise nicht angegeben. |
| selektives Berichten zu Endpunkten (reporting bias) | niedrig | Wir stuften dies als geringes Risiko für Verzerrungen ein. |
| andere Ursachen für Bias | niedrig | Wir stuften dies als geringes Risiko für Verzerrungen ein. |

| **West 1998** | | |
| --- | --- | --- |
| Methode | Prospektive, randomisierte, kontrollierte Split-Side-Studie | |
| Teilnehmende | - Patienten mit Gefäßveränderungen des Gesichts oder der Beine - Gesamt n = 8 (Gesicht), n = 12 (Bein); 1 Abbruch (Bein) wegen Schmerzen - Mittleres Alter: 40 Jahre (23–69) - Hauttyp nach Fitzpatrick: I–III | |
| Intervention | A: FSL  B: KTP-Laser | |
| Endpunkte | - Nachbeobachtung: Woche 4, 8, 12, 24; 8 und 12 Wochen nach Behandlung. - Schmerz (0–10 Skala) - Clearance (5-Punkte-Skala) - Nebenwirkungen | |
| Weiteres | Parameter:   - 1-2 Sitzungen im Abstand von 8 Wochen - Gesicht: FSL 15 J/cm²; 1,5 ms; 2 × 7 mm Spot - KTP-Laser: 15 J/cm²; 10 ms; 1 mm Spot | |
| **Risk of bias** | | |
| **Bias** | **Urteil der Autoren** | **Begründung für das Urteil** |
| Generierung der Randomisierungssequenz (selection bias) | unklar | Keine Informationen verfügbar. |
| verdeckte Gruppenzuteilung (selection bias) | unklar | Keine Informationen verfügbar. |
| Verblindung von Teilnehmern und Studienpersonal (performance bias) | unklar | Keine Informationen verfügbar. |
| Verblindung der Endpunkterhebung (detection bias) | niedrig | Ein Arzt, eine Krankenpflegerin und der Patient bewerteten das Ergebnis in einer verblindeten Weise. |
| unvollständige Daten zu Endpunkten (atrition bias) | hoch | Die Ergebnisse der Teleangiektasien im Gesicht und an den Beinen wurden teilweise nicht differenziert. |
| selektives Berichten zu Endpunkten (reporting bias) | niedrig | Wir stuften dies als geringes Risiko für Verzerrungen ein. |
| andere Ursachen für Bias | niedrig | Wir stuften dies als geringes Risiko für Verzerrungen ein. |

| **Yang 2023** | | |
| --- | --- | --- |
| Methode | Prospektive, randomisierte, kontrollierte, einfach verblindete Parallelgruppen-Studie | |
| Teilnehmende | - Patienten mit moderater bis schwerer Rosazea - Gesamt n = 44; weiblich n = 34; 3 Abbrüche (2 ALA-PDT, 1 Minocyclin) - Mittleres Alter: 34 Jahre (28–45) - Hauttyp nach Fitzpatrick: keine Angabe | |
| Intervention | A: 5-Aminolävulinsäure-Photodynamische Therapie (ALA-PDT)  B: Minocyclin | |
| Endpunkte | - Nachbeobachtung: 12 und 24 Wochen nach Behandlung. - Läsionszahl - Rosazea-spezifischer Lebensqualitäts-Score - Ärztliche Gesamtbewertung - Erythembewertung - Demodex-Dichte (Reflektanzkonfokalmikroskopie) - Rückfallrate - Nebenwirkungen | |
| Weiteres | Parameter:   - ALA-PDT: 3-5 Sitzungen im Abstand von 1-2 Wochen; 5 % ALA für 30 Min; 633 ± 10 nm rotes Diodenlicht; 30 mW/cm² für 1 Stunde - Minocyclin: 100 mg täglich für 8 Wochen | |
| **Risk of bias** | | |
| **Bias** | **Urteil der Autoren** | **Begründung für das Urteil** |
| Generierung der Randomisierungssequenz (selection bias) | niedrig | Software-Generator |
| verdeckte Gruppenzuteilung (selection bias) | unklar | Keine Informationen verfügbar. |
| Verblindung von Teilnehmern und Studienpersonal (performance bias) | unklar | Aufgrund der Art des Studiendesigns war eine Verblindung der Probanden oder des Personals nicht möglich. |
| Verblindung der Endpunkterhebung (detection bias) | niedrig | Drei verblindete Ärzte bewerteten das Ergebnis. |
| unvollständige Daten zu Endpunkten (atrition bias) | niedrig | Wir stuften dies als geringes Risiko für Verzerrungen ein. |
| selektives Berichten zu Endpunkten (reporting bias) | niedrig | Wir stuften dies als geringes Risiko für Verzerrungen ein. |
| andere Ursachen für Bias | niedrig | Wir stuften dies als geringes Risiko für Verzerrungen ein. |
