# Supplementary material for: Laser‐ und energiebasierte Systeme zur Behandlung der Rosazea – ein systematischer Review mit Netzwerk‐Metaanalyse
Source: J Dtsch Dermatol Ges. 2026 Jan 14;24(1):24–33. [Article in German] doi: 10.1111/ddg.15961_g (PMC12800870; doi:10.1111/ddg.15961_g)
Supplement: Supplementary file 3 — Supplementary information [file DDG-24-24-s003.docx]

**Charakteristika der ausgeschlossenen Studien [geordnet nach Studien-ID]**

| **Studie** | **Grund des Ausschlusses** |
| --- | --- |
| Bao 2022 | Nicht kontrolliert |
| Bennardo 2022 | Nicht kontrolliert |
| Bernstein 2008 | Nicht kontrolliert |
| Bernstein 2018 | Nicht kontrolliert |
| Bernstein 2022 | Nicht kontrolliert |
| Bernstein 2023 | Nicht kontrolliert |
| Clark 2002 | Nicht kontrolliert |
| Fan 2018 | Not controlled, data of acne vulgaris and rosacea were not differentiated |
| Kim 2021 | Nicht kontrolliert |
| Kwon 2018 | Nicht randomisiert |
| Liu 2014 | Nicht kontrolliert |
| McCoy 1997 | Nicht kontrolliert |
| Park 2023 | Nicht kontrolliert |
| Salem 2013 | Nicht randomisiert |
| Sun 2018 | Nicht kontrolliert |
| Wang 2021a | Nicht kontrolliert |
| Wang 2021b | Nicht kontrolliert |
| Wang 2022 | Zurückgezogen |
| Tirico 2020 | Kleine Stichprobe (n = 5) |

**Charakteristika der Studien, die noch bewertet werden müssen [geordnet nach Studien-ID]**

| **NCT00945373** | |
| --- | --- |
| Methode | Prospektive, offene Split-Face-Studie |
| Teilnehmende | Patienten mit Rosazea erythemato-teleangiectatica |
| Intervention | A: 2.5% Calciumdobesilat und FSL  B: FSL |
| Endpunkte | Investigator Global Assessment (IGA) scale |
| Weiteres | - |

| **NCT01631656** | |
| --- | --- |
| Methode | Prospektive, offene Split-Face-Studie |
| Teilnehmende | 15 Patienten mit milder bis moderater Rosazea |
| Intervention | A: Azelainsäure 15%-Gel und Nd.YAG-Laser  B: Nd:YAG-Laser Monotherapie |
| Endpunkte | - Investigator's Global Assessment - Unerwünschte Ereignisse |
| Weiteres | Letzte Aktualisierung am 12.09.2018, Abschluss der Studie im Februar 2011, Zugriff auf die Website im Dezember 2023. Noch nicht veröffentlicht. |

| **NCT02075671** | |
| --- | --- |
| Methode | Prospektive, randomisierte, doppelblinde Parallelgruppenstudie |
| Teilnehmende | Patienten mit Rosazea papulo-pustulosa |
| Intervention | A: 5-Aminolävulinsäure-Photodynamische Therapie  B: Vehikel und photodynamische Therapie  C: Vehikel |
| Endpunkte | - Verbesserung der entzündlichen Läsionen (Papeln, Pusteln, Knötchen), Erytheme und Teleangiektasien anhand der Investigator's Global Assessment (IGA) und der Investigator's Global Assessment für entzündliche Läsionen (ILIGA) - Klinische Erythem-Bewertungsskala (CEA) - Unterschied in der Anzahl entzündlicher Läsionen (ILC) - Gesamtbewertungsskala für Patienten (POA) |
| Weiteres | - |

| **NCT02204254** | |
| --- | --- |
| Methode | Prospektive, offene, randomisierte Studie |
| Teilnehmende | Patienten mit Rosacea papulo-pustulosa |
| Intervention | A: Radiofrequenz  B: 100 mg Doxycyclin |
| Endpunkte | - Schweregrad der Rosazea auf einer Skala von 0 bis 3 (Physician Global Assessment) - Anzahl der entzündlichen Läsionen - Patientenzufriedenheit - Dichte von Demodex |
| Weiteres | - |

| **NCT02268474** | |
| --- | --- |
| Methode | Prospektive, randomisierte, kontrollierte Split-Face-Studie |
| Teilnehmende | Patienten mit with Rosacea erythemato-teleangiectatica oder papulo-pustulosa |
| Intervention | A: 532 nm KTP-Laser  B: FSL |
| Endpunkte | Verbesserungsgrad auf einer 4-Punkte-Skala |
| Weiteres | - |

| **NCT03424304** | |
| --- | --- |
| Methode | Prospektive, offene Split-Face-Studie |
| Teilnehmende | Patienten mit Rosazea, Falten und Narben (keine weiteren Details angegeben) |
| Intervention | A: Cutera excel V™-Laser und Micro-Lens Array-Aufsatz für das Genesis V-Handstück  B: Cutera excel V™-Laser Green Genesis V-Handstück für das CoolView-Handstück |
| Endpunkte | Global Aesthetic Improvement Scale |
| Weiteres | - |

| **NCT05771298** | |
| --- | --- |
| Methode | Offene, prospektive Parallelgruppenstudie |
| Teilnehmende | Patienten mit Rosacea erythemato-teleangiectatica oder papulo-pustulosa |
| Intervention | A: 532 nm KTP-Laser  B: FSL |
| Endpunkte | - Reduktion des Erythems auf einer 5-Punkte-Skala - Schmerzintensität anhand der numerischen Bewertungsskala - Schwellung auf einer 6-Punkte-Skala - Purpura auf einer 6-Punkte-Skala - Patientenzufriedenheit auf einer 6-Punkte-Skala |
| Weiteres | - |

**Charakteristika von laufenden Studien [geordnet nach Studien-ID]**

| **ChiCTR2000038936** | |
| --- | --- |
| Titel | The efficacy and safety of pulsed dye laser in the treatment of rosacea |
| Methode | Prospective, parallel group study |
| Teilnehmende | Patients with rosacea |
| Intervention | A: PDL  B: IPL |
| Endpunkte | - clinician improvement assessment - patient global improvement - Dermatology quality of life - Transeipdermal water loss - Erythema index - Melanin index - Pain intensity on a Visual Analog Scale |
| Starttermin | 2020-10-12 |
| Kontaktdaten | Shuxian Yan  12 Middle Wulumuqi Road, Jing'an District, Shanghai 200032, China |
| Weiteres | - |

| **ChiCTR2100042816** | |
| --- | --- |
| Titel | Clinical observation of 595nm dye laser in the treatment of rosacea using different sizes of light spot |
| Methode | Prospektive Parallelgruppenstudie |
| Teilnehmende | Patienten mit Rosazea |
| Intervention | A: FSL mit 10 mm Spot  B: FSL mit 7 mm Spot |
| Endpunkte | - Erythemwert |
| Starttermin | 01.03.2021 |
| Kontaktdaten | Di Wu  120 Guanghua Street, Wuhua District, Yunnan, China |
| Weiteres | - |

| **ChiCTR2200066451** | |
| --- | --- |
| Titel | The effect evaluation of three types laser treatment for rosacea randomized trial |
| Methode | Prospektive, randomisierte Parallelgruppenstudie |
| Teilnehmende | Patienten mit Rosazea |
| Intervention | A: IPL  B: DPL  C: Elos |
| Endpunkte | - Erythem-Index |
| Starttermin | 11.12.2022 |
| Kontaktdaten | Chao Yuan  6A Build , 1278 Baode Road, Jingan District, Shanghai 200443, China |
| Weiteres | - |

| **ChiCTR2300074594** | |
| --- | --- |
| Titel | The efficacy and safety of minocycline combined with radiofrequency induction of compound lidocaine cream in the treatment of rosacea |
| Methode | Prospektive, kontrollierte Parallelgruppenstudie |
| Teilnehmende | Patienten mit Rosazea mit Symptomen im Gesicht sowie Juckreiz, Brennen, Kribbeln und Schwellungen |
| Intervention | A: Minocyclin mit Radiofrequenzinduktion von Lidocain-Creme  B: Radiofrequenzinduktion von Lidocain-Creme  C: Minocyclin mit Radiofrequenzinduktion von physiologischer Kochsalzlösung  D: Radiofrequenzinduktion von physiologischer Kochsalzlösung |
| Endpunkte | - Symptomlinderungsrate - Gesamtbewertung durch den Prüfer - Dermatologischer Lebensqualitätsindex - Rosazea-Lebensqualitätsindex - Dermatoskopie - Red-Zone-Score von VISIA - Transepidermaler Wasserverlust, Feuchtigkeitsgehalt der Hornschicht |
| Starttermin | 14.08.2023 |
| Kontaktdaten | Li Xie  West China Hospital, Sichuan University, Chengdu, China |
| Weiteres | - |

| **ChiCTR2300076423** | |
| --- | --- |
| Titel | A non-inferior randomized controlled study on the efficacy and safety of daylight photodynamic therapy (DL-PDT) for papulopustular rosacea (PPR) |
| Methode | Prospektive, randomisierte, kontrollierte Parallelgruppenstudie |
| Teilnehmende | Patienten mit Rosazea papulo-pustulosa |
| Intervention | A: Daylight photodynamische Therapie (DL-PDT)  B: konventionelle photodynamische Therapie (c-PDT)  C: orales Minocyclin-Hydrochlorid |
| Endpunkte | - Investigator’s Global Assessment (IGA) - Hautläsionswerte - clinician’s Erythema Assessment (CEA) - Lebensqualität anhand des Rosazea-spezifischen Fragebogens zur Lebensqualität (RosaQoL) - Dermatoskop - Reflektionskonfokalmikroskopie |
| Starttermin | 09.10.2023 |
| Kontaktdaten | Jian Li  Gaotanyan Main Street 30, Shapingba District, Chongqing,China |
| Weiteres | - |

| **NCT05360251** | |
| --- | --- |
| Titel | Pulsed dye laser and intense pulsed light configured with different wavelength bands in improving erythematotelangiectatic rosacea |
| Methode | Prospektive, randomisierte, kontrollierte, einfach-blinde Parallelgruppenstudie |
| Teilnehmende | Patienten mit Rosazea erythemato-teleangiectatica |
| Intervention | A: FSL  B: IPL (Delicate Pulsed Light)  C: IPL (M22 590)  D: IPL (M22 vascular filter) |
| Endpunkte | - Symptome der Rosazea (Rötung, nicht-transientes Erythem, Teleangiektasien, Brennen oder Stechen, Juckreiz, Trockenheit) anhand einer 4-Punkte-Skala - VISIA-Rote-Flächen-Score - Schmerzintensität auf einer visuellen Analogskala (VAS) - Unerwünschte Ereignisse |
| Starttermin | 2022-03-27 |
| Kontaktdaten | Suiqing S Cai  The Second Affiliated Hospital of Zhejiang University of Medicine, China |
| Weiteres | - |

| **NCT05401422** | |
| --- | --- |
| Titel | Brimonidine in rosacea |
| Methode | Prospektiv, randomisiert, kontrolliert, Parallelgruppe |
| Teilnehmende | Patienten mit Rosazea erythemato-teleangiectatica |
| Intervention | A: Doxycyclin  B: Doxycyclin und FSL  C: Doxycyclin und Brimonidin |
| Endpunkte | - Clinical erythema assessment scale - Dicke der Kapillaren mittels Dermatoskop |
| Starttermin | 25.05.2022 |
| Kontaktdaten | Hagar El Sayed  Cairo University |
| Weiteres | - |

| **NCT06033352** | |
| --- | --- |
| Titel | Potassium-titanyl phosphate (KTP) laser vs KTP laser and ivermectin cream for facial rosacea (KIR) |
| Methode | Prospektive, randomisierte, kontrollierte Split-Side-Studie |
| Teilnehmende | Patienten mit Rosacea erythemato-teleangiectatica im Gesicht oder leichter Rosacea papulo-pustulosa mit permanentem Erythem |
| Intervention | A: KTP-Laser  B: KTP-Laser und Ivermectin-Creme |
| Endpunkte | - Bewertung des Erythems anhand des Normalized Erythema Index (NEI), des Skin Redness Index (SRI), Clinical Erythema Assessment (CEA) - Veränderung der Hautläsionen anhand einer 6-Punkte-Skala der Physician Global Assessment (PGA) - Patientenzufriedenheit anhand einer 9-Punkte-Visuellen Analogskala (VAS) - Bewertung von Teleangiektasien anhand einer 4-Punkte-Skala - Veränderung der Anzahl von Papeln und Pusteln - Bewertung von Schwellungen, Rötungen, Purpura - Häufigkeit von Nebenwirkungen und unerwünschten Ereignissen |
| Starttermin | 12.09.2023 |
| Kontaktdaten | Kristine Heidemeyer, MD  Department of Dermatology, University Hospital Inselspital, Bern, Switzerland |
| Weiteres | - |

| **NTR4804** | |
| --- | --- |
| Titel | Rosacea and the Subpurpuric pulsed dye laser treatment Efficacy |
| Methode | Prospektive, randomisierte, kontrollierte, einfach verblindete Parallelgruppenstudie |
| Teilnehmende | Patienten mit Rosacea erythemato-teleangiectatica mit mindestens 5 Teleangiektasien |
| Intervention | A: subpurpurischer FSL mit maximal 4 Behandlungen im Abstand von 2 Wochen  B: subpurpurischer FSL mit maximal 4 Behandlungen im Abstand von 8 Wochen |
| Endpunkte | - Fragebogens zur Lebensqualität bei Rosazea (RosaQoL) - Auswertung von Fotos anhand der Investigators Global Assessment (IGA) und der Clinician's Erythema Assessment (CEA) - Schweregrad der Teleangiektasie anhand der Patient’s Global Assessment (PGA) |
| Starttermin | 01.03.2013 |
| Kontaktdaten | van der Linden, M.M.D.  Department of Dermatology Academic Medical Center Meibergdreef 9, 1100 DD Amsterdam, The Netherlands |
| Weiteres | - |
