# Supplementary figures and images for: Laser‐ und energiebasierte Systeme zur Behandlung der Rosazea – ein systematischer Review mit Netzwerk‐Metaanalyse
Source: J Dtsch Dermatol Ges. 2026 Jan 14;24(1):24–33. [Article in German] doi: 10.1111/ddg.15961_g (PMC12800870; doi:10.1111/ddg.15961_g)

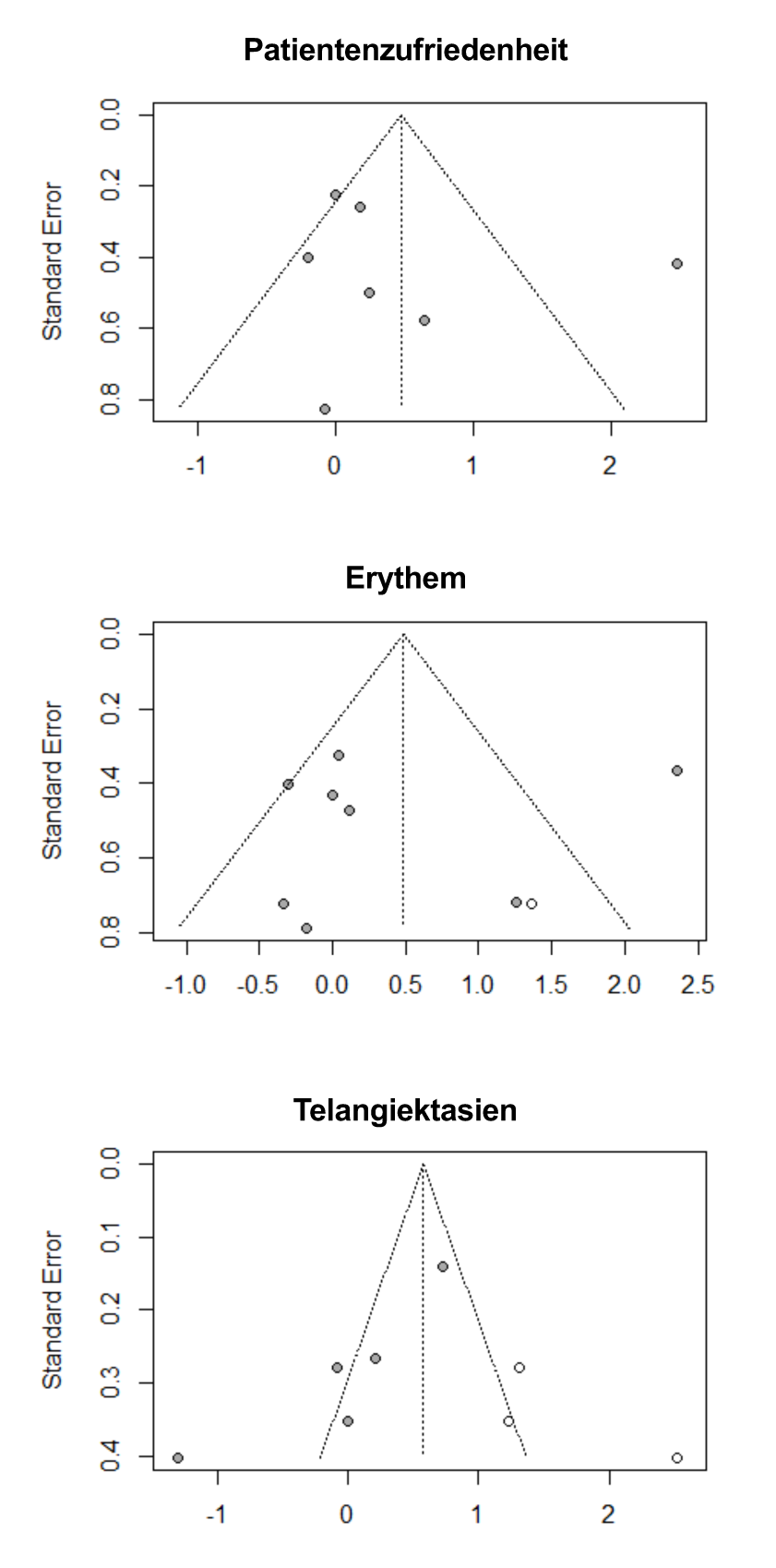

Supplement: Supplementary file 4 — Supplementary information [file DDG-24-24-s001.tif]

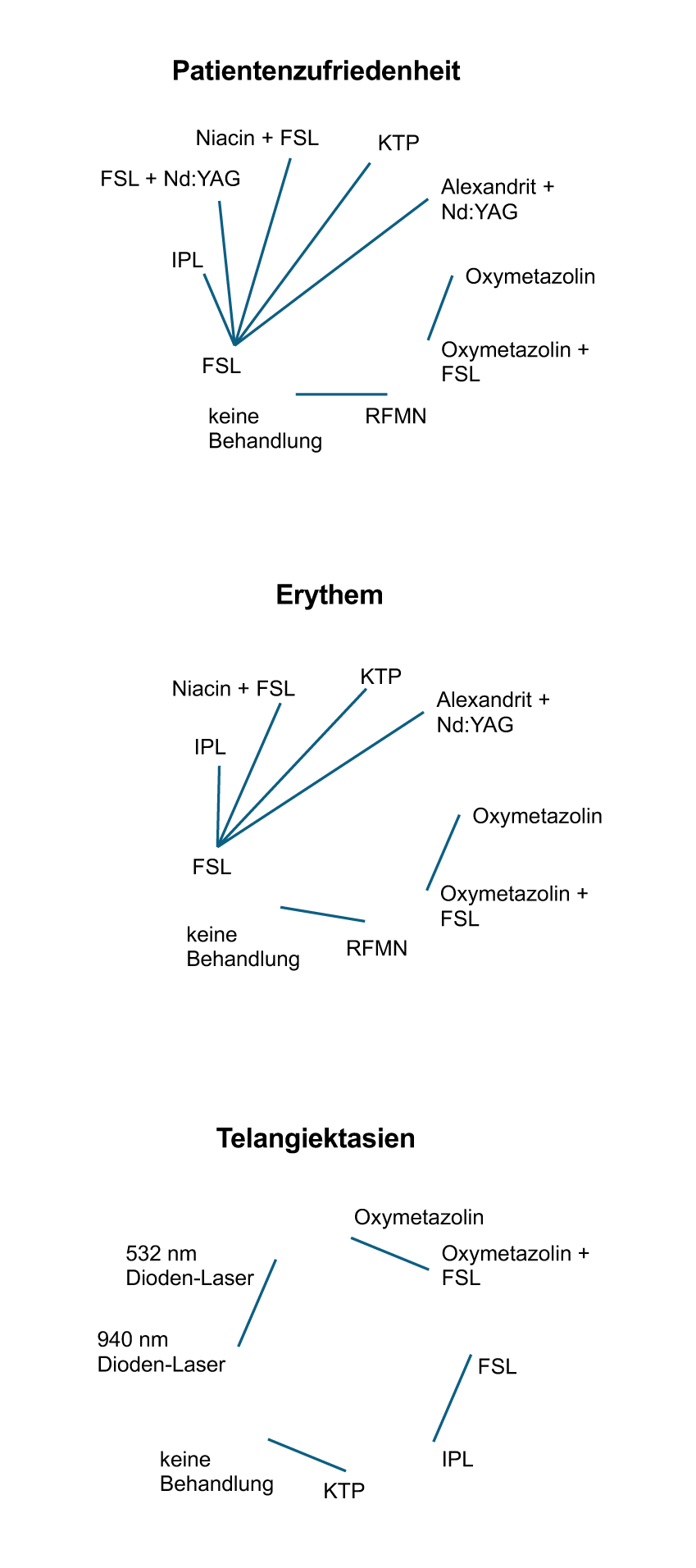

Supplement: Supplementary file 5 — Supplementary information [file DDG-24-24-s004.tif]
